# Exploring interfacial dynamics in homodimeric *S*-ribosylhomocysteine lyase (LuxS) from *Vibrio cholerae* through molecular dynamics simulations†

**DOI:** 10.1039/d0ra08809a

**Published:** 2021-01-06

**Authors:** Khair Bux, Thomas S. Hofer, Syed Tarique Moin

**Affiliations:** H.E.J. Research Institute of Chemistry, International Center for Chemical and Biological Sciences, University of Karachi Karachi-75270 Pakistan tarique.syed@iccs.edu +92-21-348-19018 +92-21-99261774; Theoretical Chemistry Division, Institute of General, Inorganic and Theoretical Chemistry, University of Innsbruck Innrain 80-82 A-6020 Innsbruck Austria T.Hofer@uibk.ac.at +43-512-507-57199 +43-512-507-57102

## Abstract

To the best of our knowledge, this is the first molecular dynamics simulation study on the dimeric form of the LuxS enzyme from *Vibrio cholerae* to evaluate its structural and dynamical properties including the dynamics of the interface formed by the two monomeric chains of the enzyme. The dynamics of the interfacial region were investigated in terms of inter-residual contacts and the associated interface area of the enzyme in its ligand-free and ligand–bound states which produced characteristics contrast in the interfacial dynamics. Moreover, the binding patterns of the two inhibitors (RHC and KRI) to the enzyme forming two different enzyme–ligand complexes were analyzed which pointed towards a varying inhibition potential of the inhibitors as also revealed by the free energies of ligand binding. It is shown that KRI is a more potent inhibitor than RHC – a substrate analogue, showing correlation with experimental data. Moreover, the role of a loop in chain B of the enzyme was found to facilitate the binding of RHC similar to that of the substrate, while KRI demonstrates a differing binding pattern. The computation of the free energy of binding for the two ligands was also carried out *via* thermodynamic integration which ultimately served to correlate the dynamical properties with the inhibition potential of two different ligands against the enzyme. Furthermore, this successful study provides a rational to suggest novel LuxS inhibitors which could become promising candidates to treat the diseases caused by a broad variety of bacterial species.

## Abstract 2

To the best of our knowledge, this is the first molecular dynamics simulation study on the dimeric form of the LuxS enzyme from *Vibrio cholerae* to evaluate its structural and dynamical properties including the dynamics of the interface formed by the two monomeric chains of the enzyme.

## Graphical abstract

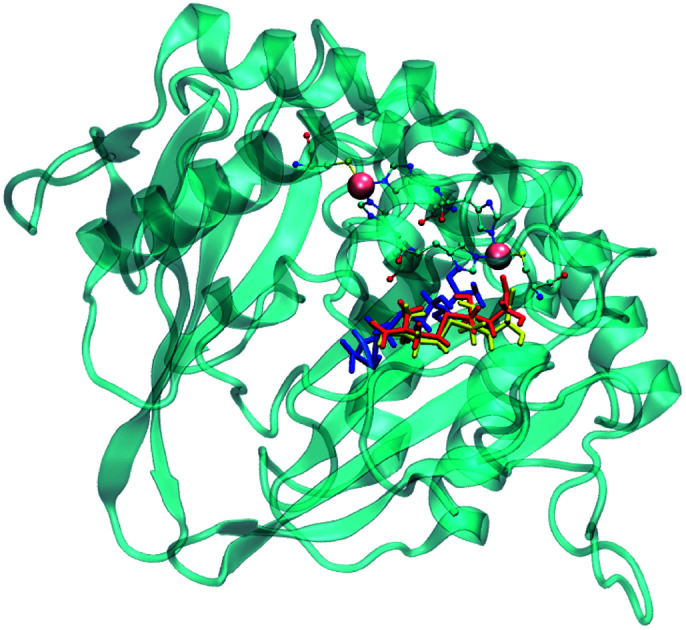

## 1 Introduction

*S*-Ribosyl homocysteinase lyase (LuxS) mediates the bacterial communication network known as quorum sensing (QS) through which most bacterial and fungal species involved in common diseases regulate their pathogenicity in terms of biofilm formation and expression of virulence factor.^1–6,78^ Typical examples include the pathogens of cholera, tuberculosis and pneumonia, as well as drug-resistant bacterial species like *Helicobacter pylori*, *Salmonellae*, and *Enterobacteriaceae* species.

QS is regulated by secretion and detection of signaling molecules known as auto-inducers (AIs) which are secreted when microbial species start accumulating around each other to a threshold level required for developing a communication network. This in turn alters the cellular density with a subsequent responsive change in their gene expression, and thus the process of communication is triggered in response to these changes in the cell density and gene expression.^7–10^ The types of QS processes were mainly categorized as either intra- or inter-species communication based on the type of signaling molecules secreted by Gram-negative and Gram-positive bacteria.^7,9,11^ The pathogenic functions like expression of virulence factors and biofilm formation in drug resistant pathogens are the result of the organized behaviour of bacterial species that is enabled through QS communication.^12–16^ The interspecies communication in Gram-negative bacteria is based on the use of acyl homoserine lactone (AHL) as AI by the bacteria. On the other hand, Gram-positive bacteria communicate by secreting auto inducing peptides (AIP) or type 2 auto-inducers (AI-2),^11,17,18^ which have been the major target of numerous studies focused on the inhibition of QS to address threats caused by lethal pathogenic bacterial species.^12,19–21^

Pathogenic Gram-positive bacteria also utilize furanosyl-borate-diester as type 2 auto-inducers (AI-2),^5,22–24^ which is catalytically synthesized through *S*-ribosylhomocysteine lyase enzyme which is also referred to LuxS. The biosynthesis of furanosyl-borate-diester begins with the conversion of a cellular methyl donor molecule commonly known as *S*-adenosylmethionine (SAM) to a toxic molecule, *S*-adenosylhomocysteine (SAH) *via* an enzyme “methyl transferase”. The SAH molecule is then converted to *S*-ribosylhomocysteine (SRH) *via* Pfs enzyme after its detoxification by removal of its adenine moiety, which is further fragmented into two molecules, homocysteine and 4,5 dihydroxy-2-3-pentadione (DPD). The latter is an unstable compound that transforms into the *R* and *S* enantiomeric furanose forms of 2-methyl 2,4 dihydroxydihydrofuran-3-one, *i.e. R*-DHMF(2*R*,4*S*) and *S*-DHMF (2*S*,4*S*).^25–27^ Considering the significant role of LuxS in the biosynthesis of AI-2(furanosyl-borate-diester), the inhibition of the enzyme has always been a major goal to take initiatives in the design of effective inhibitors against highly resistant pathogenic bacterial species like *Vibrio cholerae*.^28–32^

The LuxS enzyme is systematically known as *S*-(5-deoxy-d-ribose-5-yl)-l-homcystein-l-homcystein-lyase[(4*S*)-4,5-dihydroxy-2,3 dione and belongs to the family of carbon–sulfur lyases enzymes. A crystallographic study reported the enzyme as a homodimer with two small monomeric subunits, each consisting of 172 aminoacid residues with an identical metal-containing active site at the interface of both subunits.^33^ Structural details of the LuxS homodimer from different bacterial species revealed rare differences in the topography, *e.g.* the LuxS homodimer of *V. harvey* demonstrates high similarity with the enzymes of other sources as for instance *B. subtilis* showing exceptionally small differences in the random coil of the secondary structure elements in the aminoacid residues from 63 to 70.^34–38^ The nature of the metal in the LuxS active site was also debated for a long time, as previous studies suggested the presence of a divalent zinc ion as the metallic co-factor. However, the assumption was later rejected concluding a mononuclear divalent non-heme iron atom to be present in the catalytic site as reported through a comparative study based on the relative changes in the catalytic function with respect to variation in the metal cofactor and its stability.^35,39^ Iron was therefore found to be coordinated by three aminoacid residues His-54, His-58 and Cys-128, while the fourth coordination site of the metal was occupied by a water molecule forming the apo form of the enzyme. In case of the complex between the enzyme and a substrate/inhibitor, the water molecule was reported to be replaced with the ligand.^39^ The substitution of the coordinated water molecule with a molecular oxygen was reported to deactivate the enzyme thus resulting in the oxidation of the iron atom from Fe^2+^ to Fe^3+^.^30,39,40^

LuxS was reported to be a promising target, and initially, the main focus of studies to obtain a deeper understanding of the LuxS based QS and its inhibition.^28–32,35,39^ As far as theoretical and computational studies on the LuxS target were concerned, a combined strategy based on molecular docking and molecular dynamics (MD) simulations along with a QM/MM approach was implemented to evaluate the catalytic mechanism of LuxS.^41^ Moreover, LuxS mediated QS phenonmena in *V. harvey* were also observed to be inhibited by synthetic canabiniods.^42^ An *in silico* study consisting of molecular docking and molecular dynamics simulation was carried out to evaluate the relative inhibition potential of highly potential anti-quorum sensing lactone derivatives. Another study based on the comparative molecular similarity index analysis (COMSIA) was applied to verify the relative inhibition potential of twenty substrate analogs of ribosylhomocysteine (RHC) and other non-substrate analogs (KRI) but the study was not able to provide insights into the stability of the formed complexes at the atomistic level.^43^ Later, detailed information on the structural and dynamical properties of the monomeric LuxS enzyme complexed with the most potent RHC derivative was obtained by applying all-atom MD simulation using newly constructed force-field parameters for the iron metallocenter.^44^ The previous MD study provided detailed insight not only on the structural and dynamical properties of the enzyme and its complex with RHC inhibitor, but also revealed how the inhibitor binds to the LuxS active site. However, this study was based on the monomeric form of the enzyme and thereby neglected the role of the adjacent monomer in the substrate/inhibitor binding to the active site region located at the interface of the homodimeric enzyme. The role of the second monomer to provide additional support to the substrate was suggested to be significant for the evaluation of the interfacial dynamics required for the binding, and the stability of the substrate and/or inhibitor required to activate or inhibit LuxS, respectively.

**Scheme 1:**
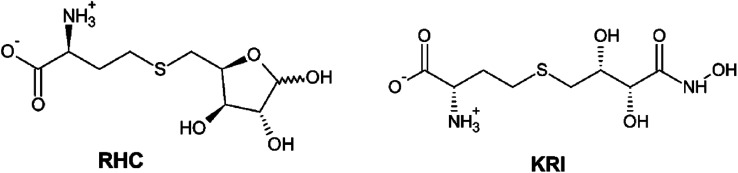
2D representation of the RHC; modified *S*-ribosylhomocysteine and KRI; (*S*)-2-amino-4-[(2*S*,3*R*)-2,3,5-trihydroxy-4-oxopentyl] thiobutanoic acid inhibitors.

Therefore, a detailed understanding of the inhibition mechanism and pattern of ligand binding as well as its effects on the structure and dynamics of the LuxS enzyme is required thereby considering the entire naturally occurring homodimeric structure of the enzyme. To achieve the objective, MD simulations were applied to the homomeric form of LuxS enzyme from *V. cholerae* complexed with a SRH analog (RHC; modified *S*-ribosylhomocysteine) as well as non-SRH analog (KRI; (*S*)-2-amino-4-[(2*S*,3*R*)-2,3,5-trihydroxy-4-oxopentyl] thiobutanoic acid) shown in Scheme 1. To the best of knowledge no complete study on the structural and dynamical features of the dimeric form of the enzyme and its complex was reported. The present study thus deals not only with the evaluation of the structural and dynamical properties of LuxS and its complexes at the atomic level, but also the dynamics of the interface region of the dimeric enzyme after complex formation with different ligands (*i.e.* substrate and non-substrate analogs) applying force-field based MD simulations. The study also focused on the structural integrity of the homodimer providing an extensive evaluation of the comparative changes in the interfacial dynamics upon ligand binding in terms of residual dynamics in both monomers, and specifically at the dimer interface. Furthermore, the study was also extended to quantify the ligand binding in terms of the associated free energy contribution to analyze the dependence of the consequent dynamical effects of the ligand binding to the LuxS enzyme involved in quorum sensing and its quenching.

## 2 Methodology

### 2.1 Protein and ligand modeling

**Fig. 1:**
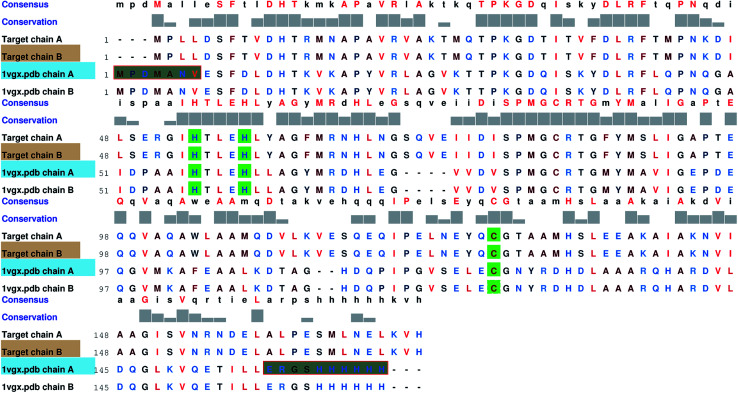
Comparsion of the sequence between the target and template enzymes (PDB ID: 1vgx); highly conserved residues are shown in green color.

Since no complete structure of the LuxS homodimer from *Vibrio cholera* was available, the protein sequence of *Vibrio cholera* (accession code: BAE87114.1)^45^ was obtained from the NCBI protein database that was then aligned with the available structures of the LuxS enzyme through BLAST,^46^ which resulted in nine similar structures with PDB IDs 1vgx, 1joe, 5v2w, xch, 1vje, 1j6x, 1j6w, 1ie0 and 5e68, however, only three protein structures “PDB ID: 1vgx, 1joe and 5v2w” with their sequence identities (greater than equal to 30 percent) with the query sequence and the remaining PDB structure had the sequence identity less than 30 percent. The query structure of the enzyme was modeled by aligning the target sequence with the template sequence which showed maximum sequence identity with the available protein structure (PDB ID: “1vgx”) (see Fig. 1) and thus the model was built using MODELLER.^47^

### 2.2 Model refinement and equilibration

The modelled three dimensional model of the enzyme was then refined *via* 3Drefine webserver.^48^ The refined model was further subjected to 50 ns MD simulation in NPT ensemble. Afterwards, the evaluation of the modelled structure was carried out based on stereochemical properties *via* the PROCHECK program.^49^

### 2.3 Active site and enzyme–inhibitor complex modeling

**Fig. 2:**
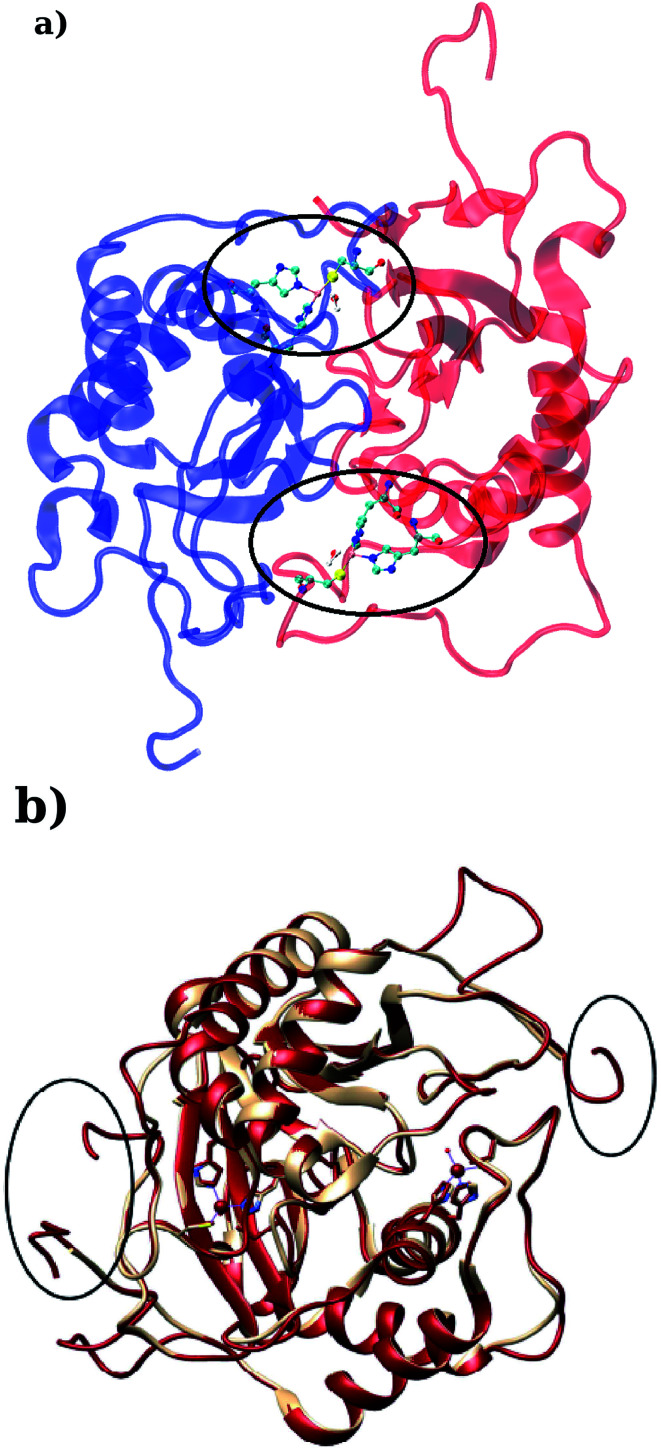
(a) Depiction of the homodimer showing the metal binding site where ligand binding takes place replacing the metal–bound water molecule where interface is formed between the two monomer (chain A; blue color and chain B; red color) and (b) superposition of the modelled enzyme (red) and the template enzyme (yellow) (PDB ID: 1vgx). Unstructured segments in the modelled enzyme were highlighted with circles.

LuxS is a homodimer enzyme and contains an identical metal centre, one of the metal center is present at the interface of the monomeric units as shown in Fig. 2a. According to the literature, the iron as ferrous ion is reported to be metal responsible for the catalytic activity of the enzyme. Therefore, iron was replaced as the metal ion in the enzyme model, and no changes in the coordination sphere (His, His, Cys and water in case of ligand-free enzyme) was reported for the iron as well. Since both metal sites in the dimer enzyme are structurally identical, the enzyme thus contains the same active site with identical metal coordination features, which enabled to implement same force field parameters reported for the metal site in the case of monomeric form of the enzyme.^44^ The binding site of the *V. cholerae* LuxS enzyme consists of a divalent Fe atom bound to three highly conserved aminoacid residues such as His-54, His-58 and Cys-128 which in the case of apo enzyme are arranged in tetrahedral fashion along with a water molecule. The latter is then substituted by inhibitors (RHC and KRI) to form the respective enzyme-inhibitor complexes. The ligand selected for the enzyme–inhibitor complex was a substrate analogue, RHC that was modelled by matching coordinates with an available co-crystallized ligand reported in the protein databank (PDB ID: 1JQW),^35^ and the ligand was further modified according to the reported chemical structure of the RHC inhibitor.^43^ According to this study, the RHC inhibitor was reported to undergo a tautomerization reaction between its active aldehydic and the associated inactive hemiacetal form, and therefore, the ligand was modified in its active aldehydic form for the manual docking.^25,50^ Another more potent inhibitor of LuxS, KRI was also modelled according to the study carried out by Vivas-Reyes and coworkers.^43^ Moreover, a constraining bond between the divalent iron and the oxygen atoms of RHC and KRI was constructed to form the enzyme–inhibitor complexes of LuxS, since the inhibitors were reported to be form complexes by coordinating to the metal ion by replacing the water molecule, thus occupying the fourth coordination site of the metallocenter in LuxS.

The modeling of ligands was carried out as all structures were modelled and optimized *via* quantum mechanical calculations with Density Functional Theory (DFT) level of theory with 6-31G** basis sets for all atoms. After the manual docking of the ligands to the metal site as mentioned above, the metal site and the surrounding coordinating amino acid residues including RHC and KRI inhibitors were minimized with the help of MOE software to remove steric clashes while the rest of the system was fixed. Afterward, ligand parameterization was performed including RESP charges derivation using generalized amber force field (GAFF) (see ESI†). It is also pertinent to mention here that the metal and the coordination residues of LuxS enzyme are conserved in all species including *B. subtilis* and *V. cholerae* whose enzyme is modelled in the dimeric form.

### 2.4 Molecular dynamics simulations

The LuxS enzyme and its complexes were simulated *via* molecular dynamics using the AMBER force-field FF14SB^51^ for the protein, while the metallocenter was treated with the force-field parameters reported in a previous simulation study conducted on the monomeric LuxS enzyme and its complex with the RHC inhibitor.^44^ The inhibitors were treated with generalized Amber force-field (GAFF) and the restrained electrostatic potential (RESP) methodology was implemented for the derivation of partial atomic charges as recommended. The protonation states of the aminoacid residues involved in the metal coordination were manually assigned and all the systems were neutralized with eight sodium (Na) ions. The systems were then solvated using a pre-equilibrated TIP3P simulation cell forming cubic boxes (total of 22 953, 26 575 and 25 585 water molecules in case of the apo enzyme, the RHC–bound enzyme and the KRI–bound enzyme, respectively) along with periodic boundary condition at a distance of ∼12 Å from each atom.^52^ In case of complexes, the NMR restraint method implemented in AMBER software includes distance, angle and torsional restraints which have been frequently reported as a reliable method for introducing the restraints in biomolecular systems. However, in the present study, the distance restraints were applied for the coordination bond between the metal ion and ligand (coordinating atoms of RHC and KRI inhibitor) at a same distance for the metal–water distance obtained from the quantum mechanical optimizations and the force constant values of 20.0 kcal mol^−1^.A were specified for the upper and lower bounds, respectively for both systems to maintain the metal–ligand coordination bond in the limit as specified for the metal–water distance. The simulation protocol is detailed in the ESI.†

#### Analysis of characteristic properties

Being a homodimer, the LuxS enzyme was assumed to display a distinct dynamical behaviour which could be better projected by estimating correlated movements of aminoacid residues, in particular those which are located near the interface of both monomer units. The ligand binding to the metallocenter located at the interface of the monomers was expected to play an important role for the evaluation of these correlative movements of the dimer. Therefore, the latter were evaluated in detail *via* their dynamic cross correlation matrix (DCCM) analysis, which was proven to be a reliable method to probe the correlated movements of aminoacid residues by projecting the entire dynamical character of proteins in the ligand-free and ligand–bound state.^56–58^ The information of the relative dynamics of the three molecular systems were obtained in terms of correlated motions by projecting the covariance matrix (σ) between two C_*α*_ atoms i and j of the enzyme from the last 10 ns of the simulation trajectories with the help of the Bio3d package implemented in the R scripting program^59,60^ based on the following equation1
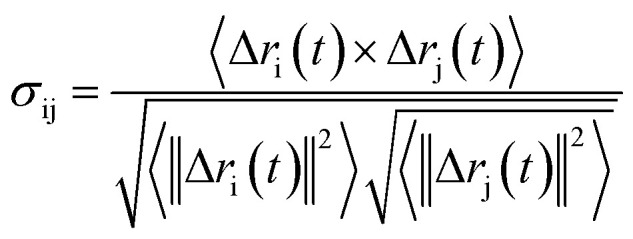
with *r*_i_(*t*) describing the projected vector of atom i as function of time, whereas ensemble time average is denoted by <‥>. The change in position of two given C_*α*_ atoms, i and j with respect to their original positions at a given time is represented as Δ*r*_i_(*t*) and Δ*r*_j_(*t*), respectively. The correlated movements projected *via* estimation matrices were subsequently visualized through two dimensional cross correlation maps which were interpreted in terms of correlation (positive) and anti-correlation (negative) in the movements employing an associated color coding in the map charts.

As mentioned the LuxS enzyme is a homodimer consisting of two interacting monomers including a metallocenter located at the respective interface, detailed understanding of the interfacial behaviour of the two monomers is essential to evaluate the inter-residual contacts. In addition, interfacial contacts (ICs) between the LuxS monomers, and inter-residual interactions at the dimer interface were evaluated through molecular dynamics consensus (MDcons). This recently introduced molecular dynamics trajectory analysis method has proven to be very reliable tool for the assessment of interfacial dynamics of homo and hetero dimer proteins or enzymes and provides complete picture of interfacial properties of the two interacting monomeric chains.^61–63^ The evaluation of the interfacial properties was based on consensus maps representing the frequency of contacts among aminoacid residues along with the rate of conservation in terms of the number of interacting residues occurring in 50, 70 and 90% of the total frames. A total of 2000 frames of the last trajectory were taken for the analysis. The criteria for the minimum distance between the two interacting aminoacid residues was set to be less than 5 Å, which was previously used in the protocol for protein analysis.^64–66^ The conservation rate of residues (CR_kl_) was taken as the ratio between the number of frames at which residues interact and the total number of frames (*N*), as shown in the following equation

2 CR_kl_ = *nc*_kl_/*N*

where *nc*_kl_ represented the total number of frames in which residue k of chain A interacted with residue l of chain B of the homodimer. Therefore, the range of conservation rate was between 1, when residues, k and l were in contact at the highest frequency in the total frames used, and 0, when residues demonstrated no contact. Similarly, the total number of conserved residues in terms of inter-residual contacts in a specific number of frames out of the total number of used frames was calculated as follows3
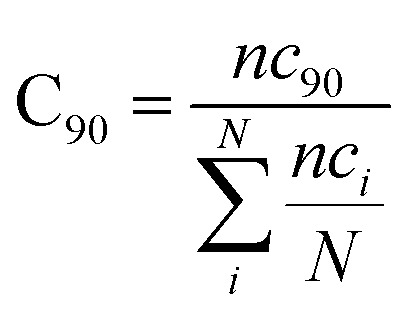
where C_90_ represented the total number of residues conserved or in the contacts that has 90% occurrence of the total frames used for the consensus analysis, while *nc*_*i*_ is the total number of residues in contact at each frame *i*.

Moreover, the estimation of the interface size of the enzyme was carried out by calculating buried accessible surface area (ASA) among the interacting aminoacid residues present at the interface of the two monomers.^61,67–69^ To study impact of inter-residual contacts on the dynamics of LuxS, the dynamical behaviour of the interfacial residues was also evaluated by means of the buried accessible surface area resulting from inter-residual contacts. The evaluation of the interface area in terms of the inter-residual contacts where both monomers form a dimer complex was carried out for LuxS in the ligand-free and ligand–bound state. By comparing the relative estimation in the influence of the ligand binding was probed, which is also thought to be associated with the overall dynamics of the enzyme. The interface area was calculated *via* the buried accessible area (ASA) between the two monomers (chain A and chain B) of the dimeric enzyme using a probe radius of 1.4 Åutilizing the NACCESS program,^69^ a frequently used tool for the interfacial analysis of protein dimers and multimers. The interfacial accessible surface area (IASA) was calculated from the buried accessible surface area(ASA), as well as the change in the accessible area (ΔASA) after the association of the two monomer chains (A and B) thus forming an interface for which the accessible area was individually computed for both chains yielding values for ASA_A_ and ASA_B_, respectively, and a subsquent calculation of the area for both chains altogether (ASA_AB_) according to the following relation.

4 IASA = ASA_chainA_ + ASA_chainB_ − ASA_AB_

### 2.5 Free energy of ligand binding

**Scheme 2:**
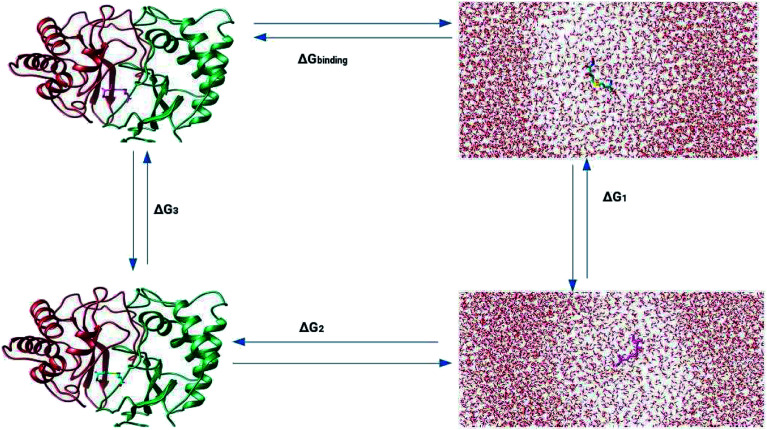
Thermodynamic cycle for the calculation of the free energy of binding in which the ligand was first assumed to be decoupled and then slowly recoupled (complexation) Δ*G*_3_, and solvated Δ*G*_1_ through the control of only van der Waals and coulombic interactions. The associated free energy of binding Δ*G*_binding_ is then estimated from the difference in the free energies of both states Δ*G*_complexation_ and Δ*G*_solvation_.

The prediction of the free energy of ligand binding to the receptor remained a challenge for the evaluation of an inhibitor potential for a specific target because of certain issues involving reliability of the method applied, accuracy and precision of the estimated results and importantly the time required for the calculation. Recent developments in free energy methods such as the advent of ensemble based molecular dynamics simulation approaches like molecular mechanics Poisson Boltzmann surface area (MM-PBSA) estimations and thermodynamic integration (TI) for the calculation of relative free energy of the ligand binding have revolutionized the field of drug development.^70,71^ In this work, the relative free energies of binding were calculated for the enzyme–ligand complexes between the LuxS enzyme, and the RHC and KRI inhibitors *via* the thermodynamic integration (TI) method. Since, the TI approach is associated to issues like ligand orientation and conformational instability with no significant change observed in the protein structure upon ligand binding, a simple thermodynamic cycle as shown in Scheme 2 proved sufficient for the estimation of the binding free energy as a difference of the free energy between the transforming states by coupling and decoupling of the ligand, Δ*G*_complexation_, with the protein/enzyme, and the solvation/desolvation of the ligand in solution, Δ*G*_desolvation_.^72,73^

5 Δ*G*_binding_ = Δ*G*_complexation_ + Δ*G*_desolvation_

as

6 Δ*G*_solvation_ = −Δ*G*_desolvation_

Thus

7 Δ*G*_binding_ = Δ*G*_complexation_ − Δ*G*_solvation_

The free energy of ligand binding was calculated by introducing a coupling parameter, *λ*, using the thermodynamic integration scheme^74^8
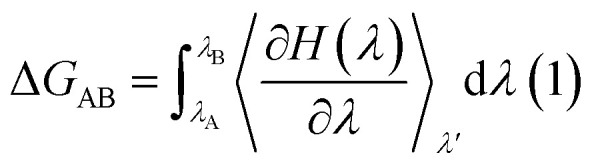
in which Hamiltonian *H* is assumed to be a function of the coupling parameter *λ via* a variation of the van der Waals interactions as well as coulombic and intra-molecular contributions by transforming the states in case of complexation (ligand–bound and ligand-free states of receptor) and ligand solvation (solvated and desolvated states). The method is based on the coupling of the ligand to the receptor *i.e.* complexation, therefore, the ligand was introduced as a dummy switching on only the van der Waals interactions (*λ* = 1) initially with gradual increase along with slowly switching off the coulombic interactions to avoid configurational instabilities. In the TI method, the estimation of the free energy solely depends on the Hamiltonian, *H* that is based on *λ*, an indicator of the variation in the ligand–protein interactions during the transformation. During the calculations, the ligand was coupled by gradual variation of *λ* between the states of coupling in which *λ* was set to 1 and decoupling corresponding to no interactions, *i.e. λ* = 0. Since TI is an ensemble molecular dynamics simulation method, a series of independent MD runs at different value of *λ* have to be performed. In each case, the gradient of the Hamiltonian, *H* with respect to the coupling variable *λ* is calculated, averaging a large number of equilibrated configurations. The subsequent integration provides an estimation of the free energy of ligand binding (Δ*G*_binding_).

Since the estimation of the free energy through the TI method solely depends on the coupling parameter *λ* as a separate degree of transformation between two states as well as the level at which Hamiltonian, *H* is perturbed, the choice of appropriately spaced *λ* points and the sampling length of the simulations are of critical importance to obtain a well-converged estimation for the Gibbs free energy of binding (Δ*G*_binding_). Therefore, coupling and decoupling of the systems in complexation and uncomplexation, solvated and unsolvated states were solely controlled *via* the coupling parameter *λ* for both Coulombic and van der Waals interactions. At the beginning, the systems were set in the state of decoupling and *λ* value was set to 0, after which the value was sequentially increased and no distance and orientational restraints were used during TI implementation. For systems in which van der Waals interactions were decoupled assuming their dominating influence in the structural organization of system, *λ* values, on other hand, were critically assigned by setting larger *λ* points along with proper clustering and random spacing mainly at the slope of the curve to obtain the actual description of the transformation taking place in the system.^72,73,75,76^ In case of protein/enzyme and other biomolecular systems, van der Waals interactions are mainly the dominating intermolecular interactions,^70,72,73^ however, considering the complexity of the systems which also involved the charge residues, thus the contribution of coulombic interactions are also significant for their conformational integrity. Therefore, the estimation of the free energy estimation of the systems was carried out by considering both the van der Waals and Coulombic interactions *via* controlling the coupling parameter *λ* at a total of 55 *λ* points in which a regular *λ* spacing was set from 0 to 0.6 with a difference of 0.02 along with a subsequent clustering with random differences of 0.01, 0.02 and 0.03 from 0.6 to 1 for each *λ* point averaged over the course of ∼1 ns MD simulations in the isothermal–isobaric ensemble. Free energy simulation protocol is provided in the ESI.†

## 3 Results and discussion

### 3.1 Homology modeling

Based on the BLAST search, the profiling of the target sequence of LuxS from *Vibrio cholera* revealed mainly three major template structures showing the highest sequence identity namely PDB IDs, 1vgx,^36^1joe^77^ and 5v2w^77^ with yielding a sequence identities of 62, 54 and 30%, respectively. The template structure 1vgx which demonstrated the highest sequence identity of 62% was selected for the modeling of the enzymatic homodimer using the multichain module of the MODELLER software.^47^ The template structure was found to have a lower number of aminoacid residues than the modeled enzyme having 172 residues in each chain of the dimer. Next, the quality of the modeled enzyme was assessed *via* Ramachandran plot (see Fig. S1†) obtained using the PROCHECK program^49^ in which the model was shown to contain 86.2% aminoacid residues falling in the favorable region along with 12.5% in the allowed and only 0.7% in the disallowed region (see also ESI†). It should also be mentioned that no information about the terminal aminoacid residues were available, resulting in unstructured terminal regions highlighting in Fig. 2b.

### 3.2 Molecular dynamics simulation

**Fig. 3:**
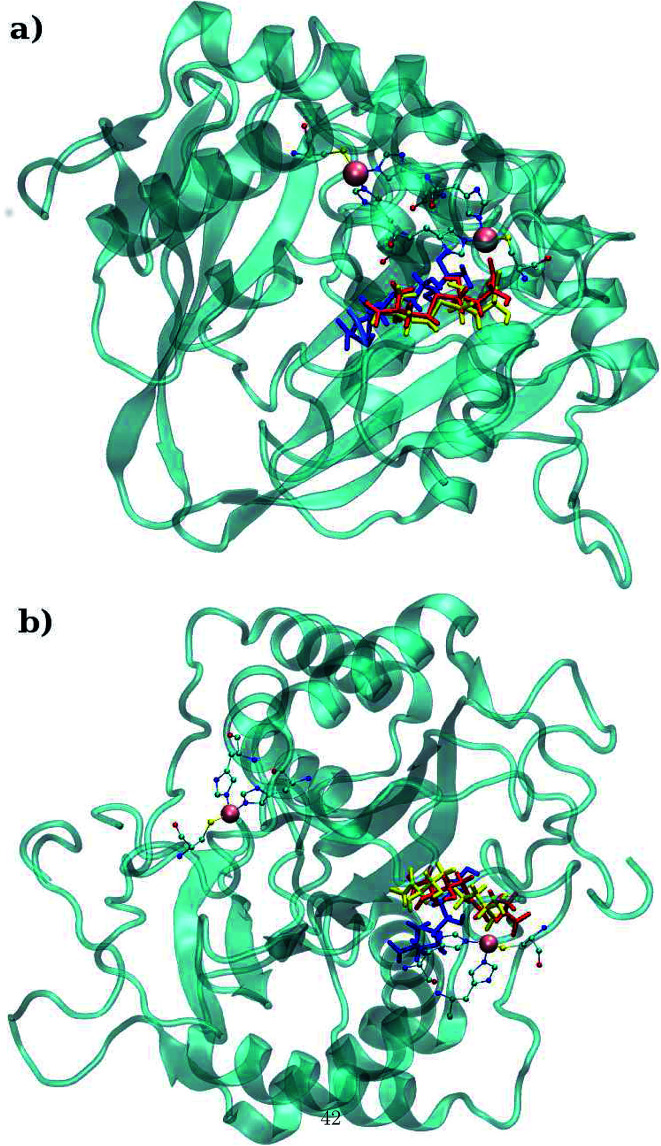
(a) Depiction of the binding mode of (a) RHC and (b) KRI to the metal containing active site of the enzyme (color codes: blue; original conformation, red; equilibrated conformation and yellow; final conformation).

**Fig. 4:**
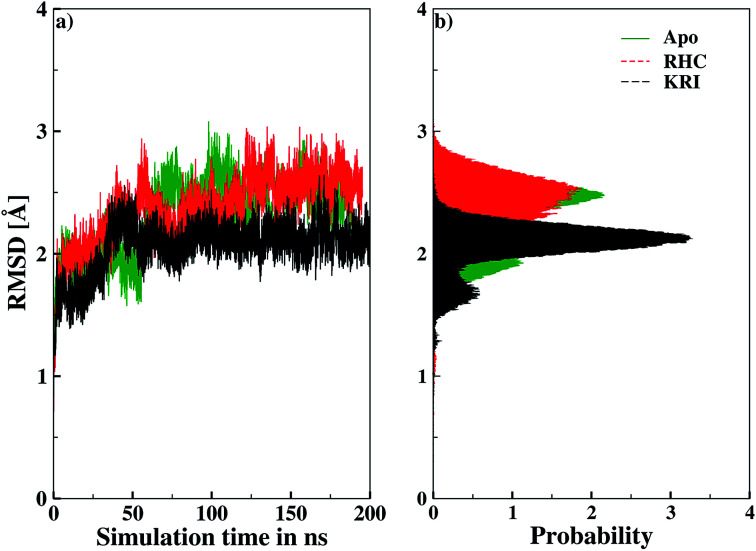
Root mean square deviation (RMSD) depicted as (a) a function of simulation time and (b) in form the respective probability plot.

**Fig. 5:**
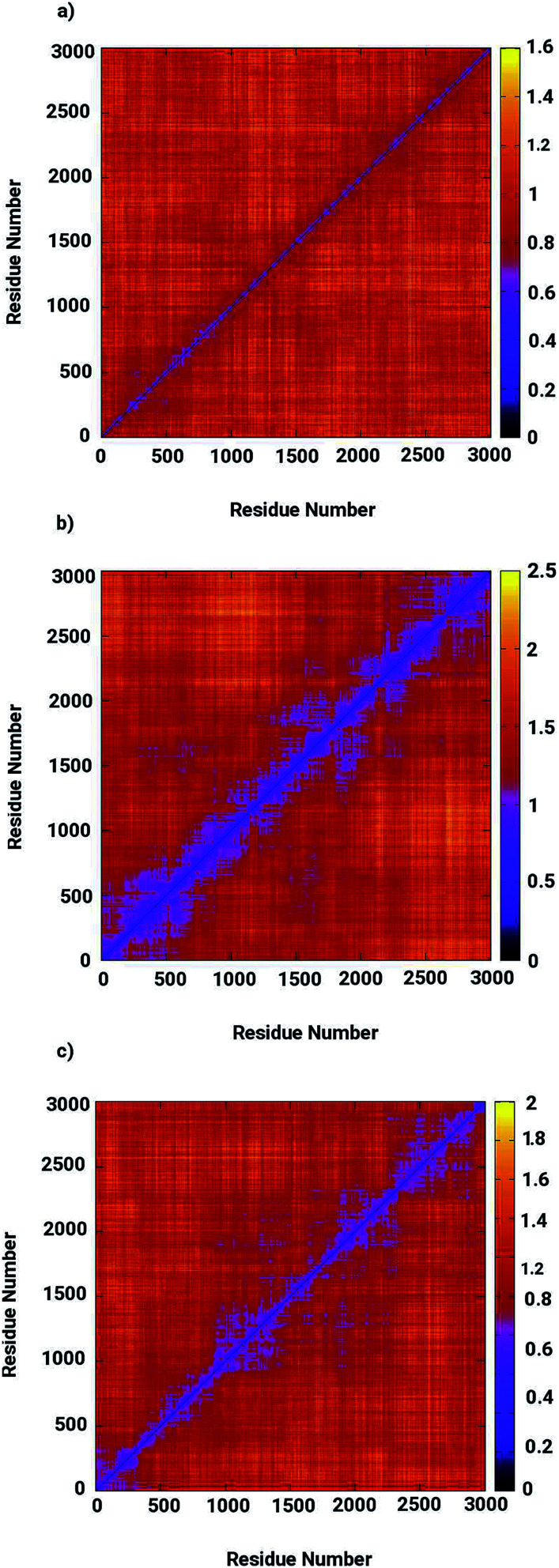
Two-dimensional root means square deviation (2D-RMSD) for (a) the ligand-free (b) the RHC–bound, and (c) the KRI–bound LuxS enzyme.

Molecular dynamics simulations were successfully implemented to study the LuxS dimeric enzyme and its two complexes with the RHC and KRI inhibitors employing the previously reported force-field parameters for the iron-containing metallocenter forming the binding site region for the substrates/inhibitors.^44^Fig. 3 illustrated the conformational modifications of the ligands before, during and after the simulations. The conformational stability of all the three systems was evaluated using root mean square deviation (RMSD) of entire trajectory run for the heavy atoms of the enzyme as a function of simulation time. Fig. 4 shows the RMSD plot for the ligand-free enzyme which fluctuates about a mean values of 2.05 ± 0.09 Å that was increased to 2.37 ± 0.11 and about 2.30 ± 0.12 Å upon RHC and KRI binding, respectively, which reflects conformational modifications of the enzyme after complex formation. The enzyme-RHC complex resulted in larger fluctuations in the RMSD compared to its KRI counterpart, which points towards a distinct binding pattern of the inhibitors. To provide further details in the conformational analysis, two-dimensional root mean square deviation (2D-RMSD) plots were computed for all the three systems shown in Fig. 5, which demonstrated a similar trend in the plots/maps with some noticeable differences in the color patterns indicating different types of conformations generated during the simulations. The ligand-free LuxS enzyme was shown to produce a lower number of distinct conformations than the ligand–bound enzyme as deduced from the 2D-RMS plots, which were further examined to distinguish conformational changes occurring after RHC and KRI binding. Based on the 2D-RMSD plots, the RHC–bound enzyme was found to be more stable compared to the KRI case, since a lower number of conformational transitions or interconversions between conformations occurred in the RHC–LuxS complex.

**Fig. 6:**
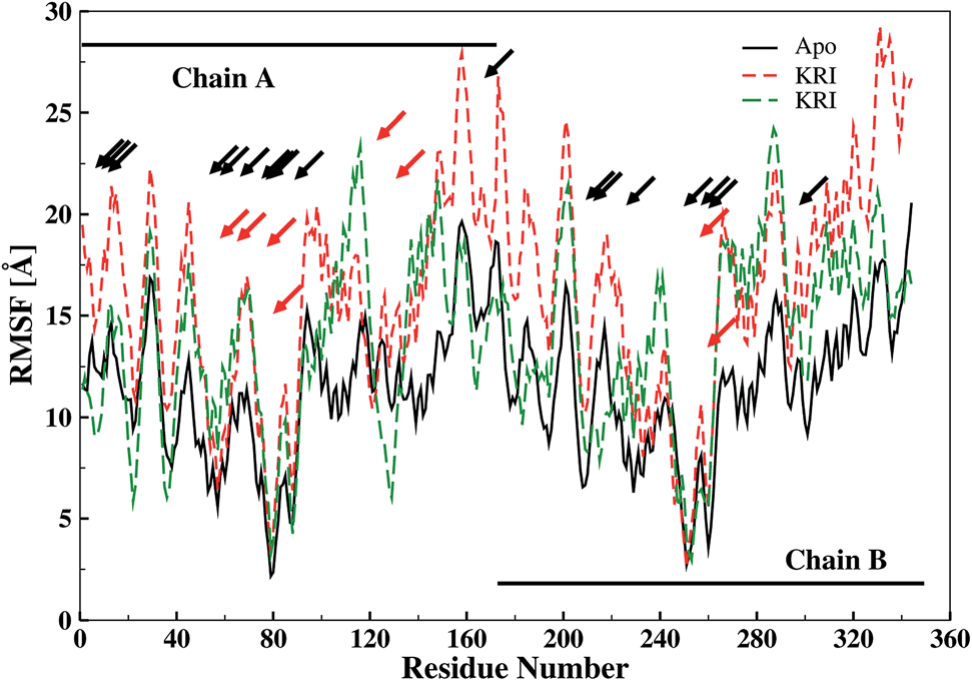
Averaged root mean square fluctuation (RMSF) of LuxS depicting the RMSF plots for both chains (monomers) of the enzyme; black and red arrows indicate the interfacial residues and ligand binding residues, respectively.

The ligand binding effect in case of the LuxS enzyme during and after complex formation encouraged to further evaluate the enzyme's dynamical flexibility in its ligand-free and ligand–bound states as well as the relative difference of dynamics between the two complexes using an averaged root mean square fluctuation (RMSF) analysis. As discussed earlier in context of the dimeric form of the enzyme and the location of the active site, the ligand accommodated at the interface region has a strong influence on the topology of LuxS as demonstrated by the averaged root mean square fluctuation (RMSF) plots depicted in Fig. 6. The ligand-free state of the enzyme shows an overall large fluctuation in the regions from aminoacid residues 115 to 165 of chain A, and from 68 to 162 of chain B relatively large fluctuations with averaged RMSF values of 11.50 Å. For the case of the complexes, the residual dynamics of the enzyme was observed to be perturbed by the ligands, since the average RMSF values for the enzyme were computed as 13.81 and 16.20 Å in the KRI and RHC case, respectively. The RMSF pattern for the case of KRI was very much similar to that of the ligand-free state in particular for the same aminoacid residues, however, residues involving the binding region showed increased fluctuations. On the other hand, the RMSF for the RHC bound enzyme demonstrates a strongly contrasting behavior, since residues of chain A display lowered fluctuations (except the last residue of the terminal region from 115 to 165 that were found to show a large dynamical flexibility), whereas chain B underwent a relatively large dynamical shift as deduced from the RMSF plot showing overall large residual fluctuations. The dynamics associated to the RHC–enzyme complex demonstrate a contrasting behavior, which points towards a distinct mode of the ligand binding into the active site situated at the interface of the dimeric enzyme. The averaged RMSFs are related to the *B*-factors^53^ and therefore can be used to compare the fluctuation data with *B*-factors obtained from X-ray crystallograhic data and NMR measurements. Generally, the *B*-factor is used in refining the crystal structure to reflect the displacement of an atom from its mean position in the crystal structure, but this property can also be evaluated for the simulation system and follow is the mathematical expression which relates *B*-factor with RMSF.9
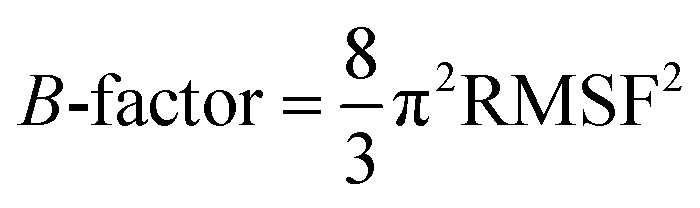


The *B*-factor the averaged *B*-factor computed for the ligand-free state of the enzyme was 2809 ± 2 Å, which is lower than that for the KRI bound enzyme (3762 ± 0.5 Å) again suggesting a strong impact of ligand binding on the structure and dynamics of the enzyme. In case of the RHC–bound LuxS enzyme, the averaged *B*-factor was found to be much higher (7651 ± 0.3 Å) than both the ligand-free and KRI bound state of the enzyme thus revealing a substantial influence on the entire dynamics of the enzyme after ligand binding which further compelled to evaluate the dynamical properties in more detail.

**Fig. 7:**
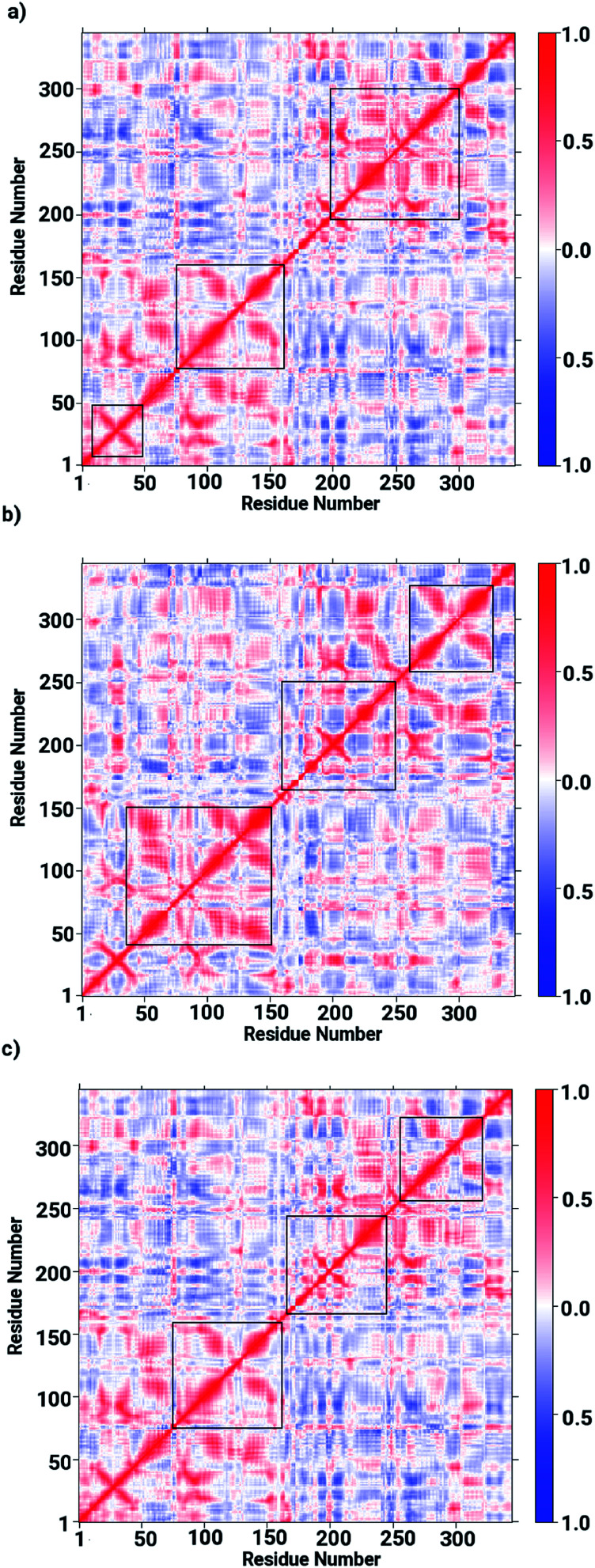
Dynamical cross correlation matrix (DCCM) maps representing inter-correlated motions for (a) the ligand-free, (b) the RHC–bound, and (c) the KRI–bound LuxS enzyme; red colored contours represent correlated movement, while the blue colored contours correspond to anti-correlated movements.

Based on the obtained data on the enzyme dynamics presented above the contrasting dynamical features after ligand binding were further explored by the evaluation of the inter-correlated domain movement in terms of the covariance matrices of the C_*α*_ atoms *via* a dynamical cross correlation matrix (DCCM) analysis. Fig. 7 depicts the DCCM plots demonstrating mixed correlation shown by positive and negative correlation maps corresponding to correlated and anti-correlated domain movement as a function of the residue number. In case of the ligand-free enzyme, positively correlated movement was expected in the initial residues of chain A (from 10 to 150 aminoacids) and in the last residues of chain B (from 230 to 240 aminoacids), however, the enzyme was found to possess anti-correlated motion in a number of regions, in particular from aminoacid residues from 150 to 230, forming the interface region involving the loops of both chains in a parallel orientation (*cf.*Fig. 7a). This anti-correlated motion is strongly linked to the region involving the metallocenter and has a strong impact on the interfacial dynamics that is also assumed to be associated with enzyme functions that are in turn perturbed by a ligand binding. The DCCM plot for the RHC bound enzyme depicted in Fig. 7b reveals substantial opposite character in the motion of the monomeric chains. While chain A demonstrates correlated movements, anti-correlated molecular motion was registered for chain B, thus revealing a dramatic change in the dynamical picture upon ligand binding. This also suggested a distinct pattern of the RHC binding at the metallocenter situated in the interfacial region where the ligand was expected to be interacting with aminoacid residues of both chains giving rise to the observed anti-correlated chain movements. In case of the KRI–bound enzyme, the cross correlation map revealed a dynamical behaviour similar to that of the ligand-free state with an expected perturbation in the overall dynamics of the enzyme after ligand binding (*cf.*Fig. 7c). However, the strong contrast in the dynamics of the RHC– and KRI–bound enzymes is visible, which correlates well with earlier findings based on the RMSF/*B*-factor analysis.

**Fig. 8:**
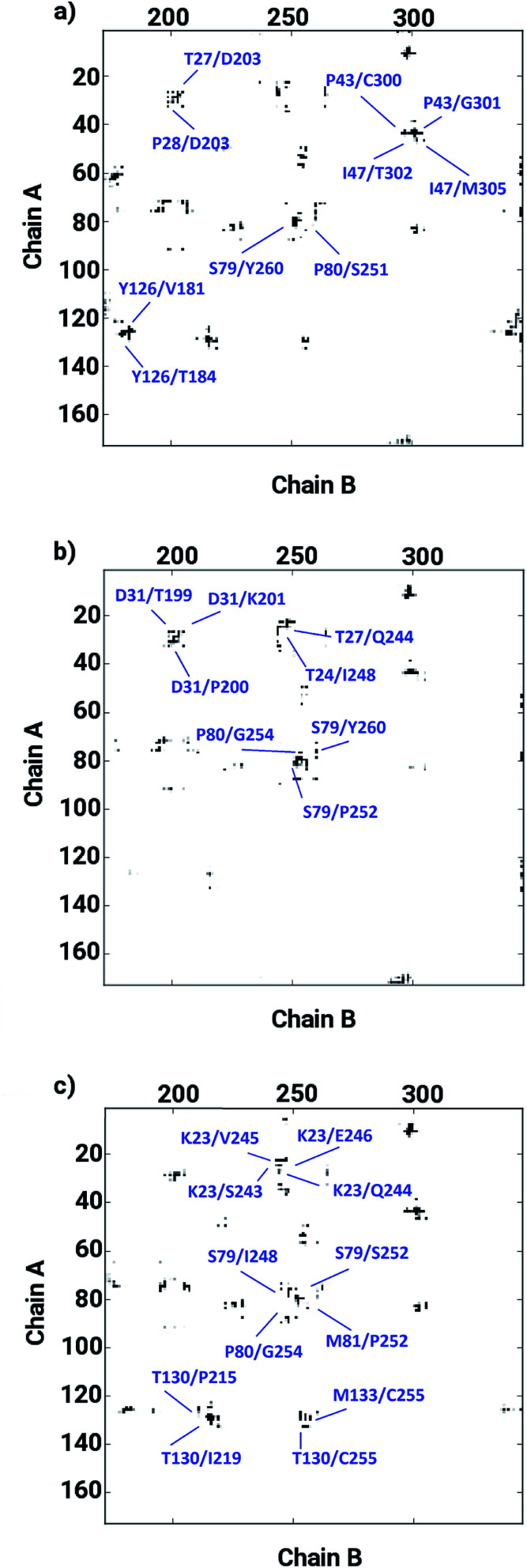
Inter-residual contacts representing the associated frequency through the respective dot density: the higher the density of dots the more contacts are present, among residues at the interfacial region of the monomeric chains in case of (a) the ligand-free, (b) the RHC–bound, and (c) the KRI–bound LuxS enzyme, respectively.

The ligand binding at the interface of the dimeric enzyme close to the metallocenter motivated a further exploration of the dynamics of the interfacial region quantifying the conservation of the structural properties in terms of residual interactions/contacts between both monomeric chains. Based on the data shown in Table 1, the ligand-free LuxS enzyme demonstrated 71% of the 130 interface residues to be conserved, and interacting between each other with a high conservation rate (CR_kl_) showing a value of one in 90% of the frames in the simulation trajectory. This implies that a large number of contacts at the interface render the system less flexible and therefore, more stable. Upon binding of RHC to the active site, the interfacial dynamics is altered showing a reduced number of interfacial residues quantified of about 67 (55% out of the total number of interfacial contacts). In case of the KRI bound–enzyme, the number of interfacial residues interacting with each other was found to be about 95 with 90% of then showing a high rate of conservation close to 1, which again can be interpreted as a low flexibility/high stability of the interfacial region compared to the RHC–bound LuxS enzyme. To further confirm the interaction between the interfacial residues across both chains and the associated conservation among the interfacial residues, the associated inter-residual contact (ICs) were analyzed in form of comparative consensus maps among the three systems based on the frequency of inter-residual contacts among residues across the two chains (see Fig. 8). Following the consensus plots, the ligand-free state of the enzyme is shown to have all interfacial residues interacting with each other across both chains resulting in low dynamics of this highly conserved interfacial region (*cf.*Fig. 8). The consensus map for the RHC–bound enzyme exhibits a reduced number of inter-residual contacts (ICs) thus revealing a low conservation rate and high flexibility of the interfacial region upon ligand binding correlating well with the earlier findings. The consensus map for the KRI–bound enzyme shows a large conservation suggesting a high degree of inter-residual contacts between the interfacial residues, which is consistent with the inter-residual contact data listed in Table 1. This also indicates a higher relative flexibility of the interfacial region in the KRI case than that observed for the RHC–bound enzyme. Clearly the differences in the binding pattern is responsible for this strong contrast in the dynamical properties of the two enzyme–inhibitor complexes.

**Table 1:** Comparison of inter-residual contacts between chain A and B, in the ligand-free, the RHC–bound and the KRI–bound enzyme, in the given 2000 frames used for the estimation of the rate of conservation frequency CR_kl_ = 1 along with the average interfacial area in Å^2^

System	C_50_	C_70_	C_90_	ICs CR_kl_ = 1	MD IASA
Apo	0.9661	0.8573	0.7151	128	17 669.5 ± 0.01015
RHC	0.9401	0.8091	0.6531	67	17 064.3 ± 0.01262
KRI	0.9862	0.8461	0.6836	95	17 082.5 ± 0.01064

**Fig. 9:**
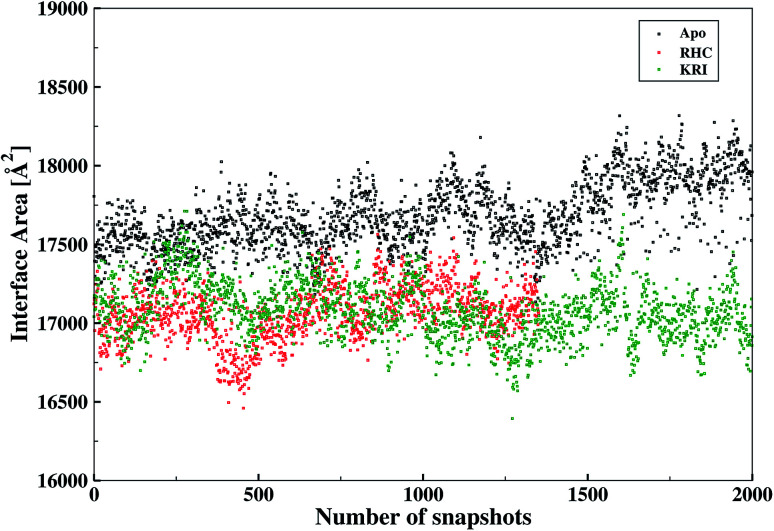
Interfacial area calculated from the buried accessible surface area (ASA) of the LuxS dimer upon interactions between its two monomeric chains, and the respective changes after binding of the ligands.

The inter-residual contacts among the interfacial residues across both chains were also expected to affect the accessible surface area (ASA) of the interfacial region. This buried ASA could also be influenced by ligand binding to the enzyme which provides additional information on the interfacial dynamics and the constrasting binding properties of the two different ligands. The interface area was therefore, calculated in terms of the buried ASA of the ligand-free and ligand–bound state of the enzyme as a function of the simulation time illustrated in Fig. 9. The ligand-free enzyme was found to have a large interfacial area of 4712.8 ± 0.02 Å^2^ indicating a more buried ASA due to the large number of interactions between the interfacial residues, that was reduced to 2168.6 ± 0.04 Å^2^ in case of the RHC–bound enzyme. This draws a clear picture of the ligand binding effect on the interface, since the number of interactions among the interfacial residues is reduced upon ligand binding. On the other hand, the averaged area for the KRI bound enzyme of 2770.83 ± 0.83 Å^2^ is higher than that of the RHC–bound enzyme, while remaining lower compared to the ligand-free state. This suggests a similar mechanism reducing the number of interactions among the interfacial residues resulting in a reduced interfacial area upon ligand binding. Nevertheless, again a contrasting behaviour of the two ligands towards the enzyme was visible from the interfacial area analysis, and the difference provides valuable information to evaluate the distinct interfacial dynamics occurring after binding of the two different ligands; RHC binding resulted in a large influence on the interface region of the enzyme as quantified by the low interfacial area as well as a low number of inter-residual contacts of the two chains thus indicating that the most probable binding of the ligand took place at the interface of both chains. Contrary, the binding of KRI was thought to take place preferentially in the proximity of only one chain, which therefore, caused less disruption of the inter-residual contacts thus resulting in a relatively high value for the interfacial area compared to the RHC case, while still remaining lower than the ligand-free state of the enzyme.

**Fig. 10:**
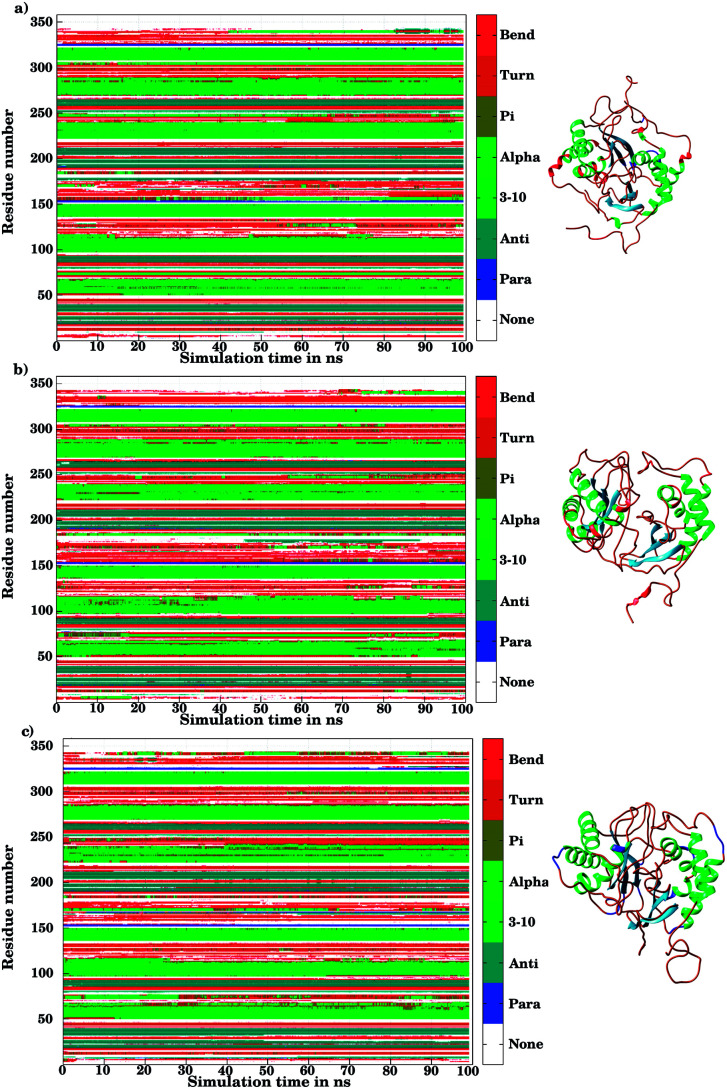
Secondary structure element analysis using the DSSP program for (a) the ligand-free, (b) the RHC–bound, and (c) the KRI–bound LuxS enzyme, respectively.

The ligand binding to LuxS caused the secondary structure elements to undergo notable conformational and structural modifications as evidenced by the DSSP plots as a function of simulation time illustrated in Fig. 10. A careful investigation of the plots revealed a perturbation in the enzymatic structure after ligation, and aminoacid residues of the binding site and its proximity experienced the ligand binding effect as they underwent transitions, for instance Glu50, Arg51 and Leu68 of the chain A, and Cys230 and Glu268 of the chain B in the coil form were significantly perturbed to attain helical form after RHC binding. Similarly, aminoacid residues of the regions from 57 to 59, from 72 to 74, from 112 to 116 including Thr97 of chain A, as well as the aminoacid residues Leu231, Ala303, Ala304, and the regions from 286 to 288, and from 338 to 340 of chain B were helical prior to ligation which were later converted to a coil motif upon ligand binding. It is noteworthy that the aminoacid residues existing in β-sheets remained mainly unperturbed after RHC binding. In case of the KRI–LuxS complex, the secondary structure elements of the enzyme were seen to be affected as β-sheets involving Glu26 and Thr32 of chain A, and Thr264 of chain B, which underwent transitions to coils after complex formation. Moreover, aminoacid residues Glu50 and Arg51 of chain A transformed from loop to helix upon ligand binding. In addition, the aminoacid residues of the regions from 72 to 74, and from 113 to 116 of chain A as well as the aminoacid residues of chain B including Asn241, Gly242, the region from 286 to 288, Cys230, Leu231, Met236, Thr268, Glu269, Ala303 and Ala304 of chain B seemed to be affected by KRI binding as these aminoacids were transformed from helical to coil motifs. The secondary structure analysis clearly shows that ligand binding resulted in significant structural and conformational modifications in both chains of the enzyme. Together with the differences observed in the interfacial dynamics of the systems these finding prompted to further investigate the binding patterns of RHC and KRI to the LuxS enzyme.

**Fig. 11:**
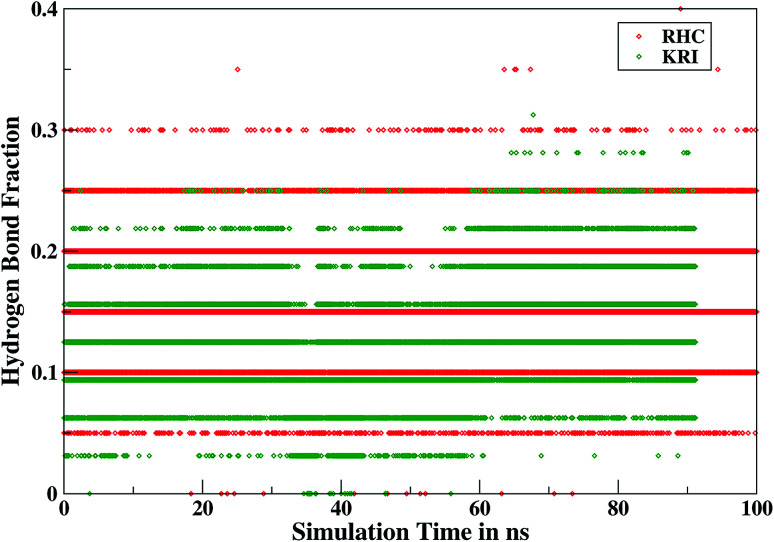
Time evolution of hydrogen bonds as a function of the simulation time for the RHC–bound and KRI–bound LuxS enzyme.

**Fig. 12:**
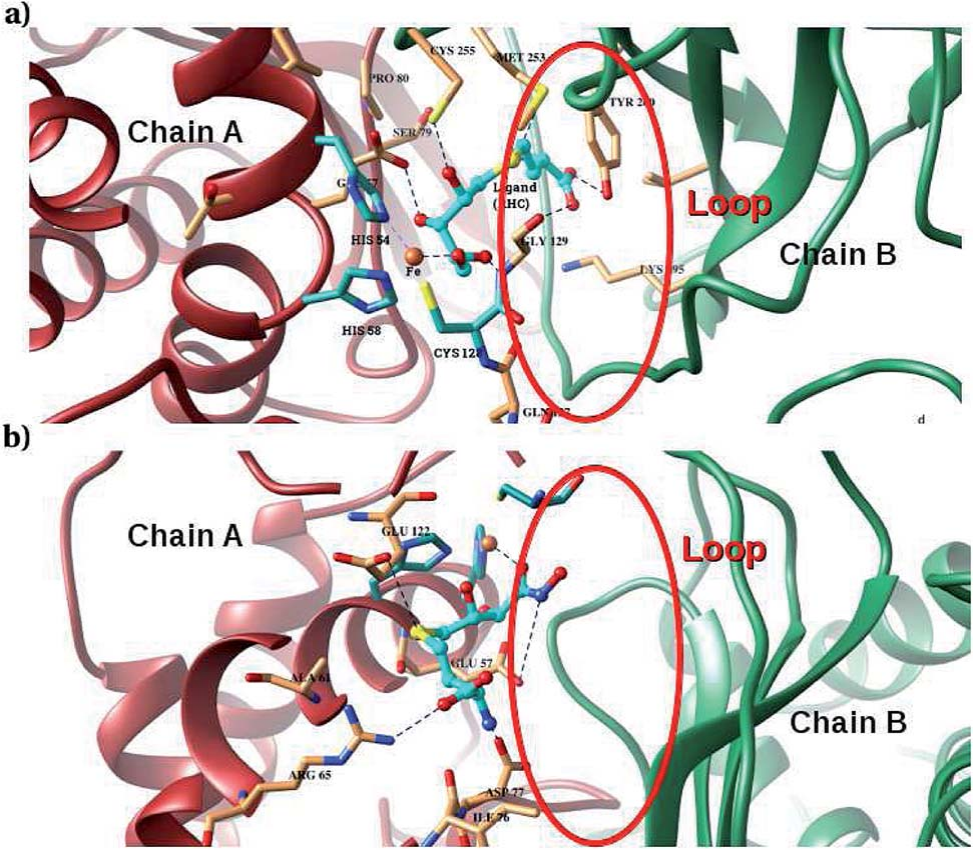
Key H-bond interactions of (a) RHC and (b) KRI with neighbouring residues of the LuxS enzyme.

Furthermore, interactions of the ligands to the LuxS enzyme were evaluated mainly *via* a hydrogen bonding analysis in terms, evaluating the fraction of hydrogen bonding as a function of simulation time shown in Fig. 11. The H-bonding fraction for the RHC–bound enzyme was found to be 0.45, which is larger than in the case of KRI yielding a H-bonding fraction of 0.25. Thus points towards an increased number of hydrogen bond interactions being formed between the enzyme and RHC. Fig. 12 displays a representative snapshot taken from the simulation trajectory depicting the interaction of the RHC inhibitor and the aminoacid residues of both chains of the LuxS enzyme *via* hydrogen bonding. Conversely, the KRI inhibitor is involved only in interactions with aminoacid residues of chain A. The hydrogen bonding analysis confirmed the difference in the binding pattern of the two ligands which were earlier shown to affect the dynamical properties of the enzyme after ligation. Since most of the residues belonging to chain A and B were involved in hydrogen bond interactions with the RHC inhibitor, in particular the loop region of chain B (except Lys195 that exists in beta sheet confirmation). On the other hand, the aminoacid residues of chain A involved in hydrogen bond interactions with the KRI inhibitor were found to be in the helical structure (except Ile76 that attained a loop structure). Based on the hydrogen bonding analysis, it was thus established that the difference in the dynamical properties of the LuxS enzyme are associated to the binding patterns of the two ligands, which belong to two different classes of chemical compounds.

### 3.3 Free energy of binding

**Fig. 13:**
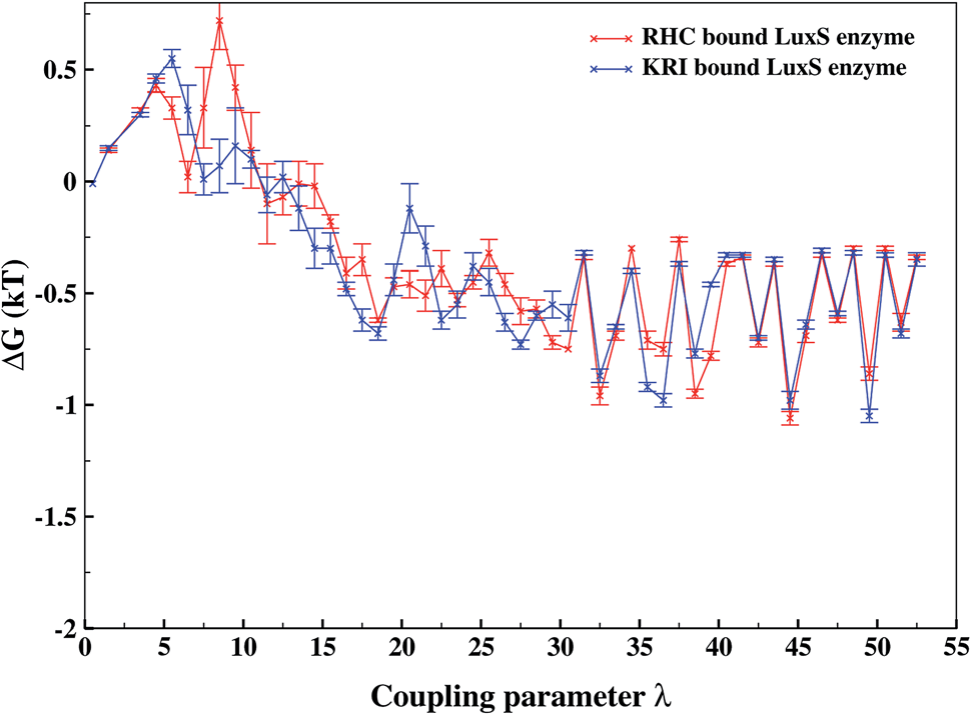
Free energy of ligand binding for the RHC and KRI inhibitors to the enzyme as a function of *λ* points obtained *via* thermodynamic integration.

As revealed by the hydrogen bonding analysis, the two ligands were found to have different binding patterns towards the enzyme, which were also presumed to have major influence on the associated binding potential. Therefore, the computation of the free energy of ligand binding was carried out employing the thermodynamic integration (TI) approach. Fig. 13 depicted the plot of the relative free energies (DG in kT units) for each interval of *λ* that is between neighboring Hamiltonian for both ligand–bound systems and the free energy change from *λ* = 0 to *λ* = 1 was simply the sum of the free energy changes of each pair of neighboring *λ* simulations. The computed binding free energy Δ*G*_bind_ for RHC- and KRI-binding to the LuxS enzyme were found to be −44.4 ± 3.2 and −47.2 ± 0.6 kJ mol^−1^, respectively, thus indicating the KRI inhibitor to have a higher potential of inhibiting the enzyme compared to the RHC case. The difference in the inhibition potential of the two ligands towards the enzyme demonstrates also that the RHC inhibitor has low potency compared to KRI inhibitor as deduced from the respective experimental *K*_i_ value of 4.2 and 0.37 μM for the RHC and KRI inhibitor towards the LuxS enzyme.^43^ Still the difference in the free energy data obtained *via* TI is approximately 10 times lower than the experimental binding constant for the two ligands, which could be inferred as the involvement of different coordinating atoms of the RHC and KRI inhibitors towards the metal ion. The coordination bonds between the inhibitors and the metal ion were treated classically by applying distance restraint during MD simulations that can not fully account the metal–ligand contribution in the free energy estimation, however the energy data are encouraging that provides information on the relative binding potential of the two ligands – the KRI inhibitor being more potent compared to RHC. The ligand affinity difference obtained *via* the TI method correlated with the reported experimental *K*_i_ values which therefore confirm the contrasting features in the structural and dynamical properties of the enzyme after the ligation by two different ligands. The energy data therefore, strengthened the observation of the distinct binding patterns of the two ligands bound to the metal-containing active site located at the interface of the two monomers, in addition to the dynamical properties evaluated so far. Table 2 lists the thermodynamic data involving the free energy of complexation and free energy of solvation, which then give the free energy of ligand binding. The difference between Δ*G*_bind_ for RHC- and KRI-binding is then given as of −2.8 kJ mol^−1^. Based on the thermodynamic data, the KRI inhibitor was found to have a higher affinity towards the enzyme compared to RHC inhibitor, which occupies the interfacial region as deduced from both the structural and dynamical data. Moreover, the evaluation of inter-residual contacts, interfacial dynamics and hydrogen bond interactions for the ligands bound to the enzyme further corroborated the Δ*G*_bind_ values correlating with the difference of the inhibition potential of the two ligands. The binding of RHC at the interface of LuxS not only enhances the flexibility of the enzyme but also the affinity of the ligand was influenced resulting in a decreased free energy value and thus inhibition potential. Moreover, the residues of the loop interacting with RHC did not contribute in enhancing the binding potential of the ligand towards the enzyme, since the loop's having high dynamical flexibility was not perturbed by ligand binding in this case. On the other hand, binding of KRI to the enzyme did not involve the interfacial residue including the critical loop region, which results in a lower dynamical flexibility and conferred the ligand to have a higher affinity towards the enzyme thus yielding a more negative value of Δ*G*_bind_.

**Table 2:** Free energies of ligand binding in kJ mol^−1^ obtained from free energy simulations using the thermodynamic integration approach

Enzyme–inhibitor complexes	Binding free energies (kJ mol^−1^)
RHC–bound LuxS enzyme	−44.4 ± 3.2
KRI–bound LuxS enzyme	−47.2 ± 0.6

## 4 Conclusion

Based on the application of MD simulations to the dimeric form of the LuxS enzyme in its ligand-free and ligand–bound states with a substrate analogue, RHC as well as a non-analogue, KRI, notable differences in the structural and dynamical properties were identified after ligation of the enzyme by these different inhibitors. The binding analysis of RHC and KRI served to explore the interfacial dynamics in detail after complex formation, since the binding took place at the metallocenter located at the interface region of the LuxS dimer. Binding of the inhibitors revealed the dynamical role of a loop structure of chain B that was reported to facilitate the binding of substrates/inhibitors. This prompted us to explore the role of the loop located in close proximity of interfacial region, and its role in stabilizing the respective ligand complex. It was found that the substrate analogue inhibitor, RHC was accommodated at the interface of the enzyme including the loop-facilitated stabilization as reported for the substrate (RSH). However, a contrasting behavior was observed in case of the non-substrate analogue KRI that was stabilized by aminoacid residues of chain A only, thus providing evidence on a missing involvement of chain B upon KRI binding, highlighting that the KRI inhibitor is not located at the interfacial region after binding to the enzyme. Moreover, the evaluation of the contrasting binding patterns of the inhibitors also aided to establish that the involvement of the loop in chain B did not contribute much to increase the inhibition potential of the enzyme as evaluated by the computed binding affinities of the ligands. This finding correlates well with experimental data reported in the literature showing the KRI inhibitor to have a higher inhibition potency than its RHC counterpart. Based on the findings, future inhibitor design against the LuxS enzyme can be rationalized with a broader perspective to pave the way to explore new quorum sensing inhibitors or quorum quenchers against infectious diseases caused by bacteria, or in particular cholera caused by *Vibrio cholerae*.

## Conflicts of interest

There are no conflicts to declare.

## Supplementary Material

RA-011-D0RA08809A-s001
