# Supplementary material for: Exploring interfacial dynamics in homodimeric S-ribosylhomocysteine lyase (LuxS) from Vibrio cholerae through molecular dynamics simulations
Source: RSC Adv. 2021 Jan 6;11(3):1700–14. doi: 10.1039/d0ra08809a (PMC8693604; doi:10.1039/d0ra08809a)
Supplement: RA-011-D0RA08809A-s001 [file RA-011-D0RA08809A-s001.pdf]

Electronic Supplementary Material (ESI) for RSC Advances.
This journal is © The Royal Society of Chemistry 2021

Exploring Interfacial Dynamics in Homodimeric S-ribosylhomocysteine Lyases (LuxS) from *Vibrio Cholerae* Through Molecular Dynamics Simulation

Khair Bux[1], Thomas S. Hofer*[2], and Syed Tarique Moin†[1]

[1] *H.E.J. Research Institute of Chemistry, International Center for Chemical and Biological Sciences, University of Karachi, Karachi-75270, Pakistan, E-Mail: tarique.syed@iccs.edu, Tel.: +92-21-99261774, Fax: +92-21-348-19018*

[2] *Theoretical Chemistry Division, Institute of General, Inorganic and Theoretical Chemistry, University of Innsbruck, Innrain 80-82, A-6020 Innsbruck, Austria, E-Mail: T.Hofer@uibk.ac.at, Tel.: +43-512-507-57102, Fax: +43-512-507–57199*

Dated: December 29, 2020

*Corresponding author
†Corresponding author

Simulation protocol: All systems were subjected to enzyme minimization steps prior to equilibration to remove close-ranged intra- as well as inter-molecular interactions using the PMEMD module implemented in AMBER18 [1, 2]. To keep the coordination bond between the iron and inhibitors, distance restraints were applied throughout the simulation time from minimization to production MD. Initially 500 steps of minimization were carried out for the water molecules while applying restraints on the enzyme using a force constant of 25 kcal.mol^{-1}.Å^{-1}. Afterwards, the force constant was gradually released after every 100 steps, thus a total of 1000 steps of restrained minimization were performed followed by an unrestrained minimization for 500 steps. After the minimization, the systems were subjected to heating and equilibration which involved thermal equilibration using the Langevin thermostat at 300 K. During the equilibration phase, pressure was maintained at $\sim$1 atm using a relexation time of 1 ps in the pressure coupling algorithm [3]. Finally, simulations for productions were performed in the isothermal-isobaric (NPT) ensemble for a total of 200 ns for all the three systems with the sampling of the simulation trajectories performed every 2 ps using PMEMD module implemented with CUDA executed with the help of GPU based workstation. The first 100 ns MD simulation was considered as equilibration stage for all the systems and the final 100 ns simulation was for production MD. During the whole simulation time, bonds involving H-atoms were constrained employing the SHAKE algorithm [3] with a time interval of 2 fs, and the long-ranged electrostatic interactions were treated by the Particle Mesh Ewald (PME) algorithm [4] with the associated cutoff distance set to 10 Å. In addition, for the case of complexes, the metal-ligand distance was attempted to keep restrained within range between 1.9 and 2.0 Å as for the metal-water

coordination distance in the ligand-free enzyme along with certain parameters for the NMR restraint method, whereas the rest of the system was set free during the whole simulation time that is during equilibration as well as the production runs (overall simulation time). The simulation trajectories were analyzed with help of the cpptraj module implemented in AMBERTOOLS18 [5, 1], VMD [6] and Chimera [7] were employed for the visualization of the simulation data.

Free energy simulation protocol: MD simulations for the free energy calculation were carried out for both complexes of the LuxS enzyme with the RHC and KRI inhibitors following an appropriate protocol required for the simulation at each λ point. Prior to the simulations, minimization of the systems was carried out twice, first using the steepest descent algorithm, and second the L-BFGS algorithm for the minimization in order to avoid instabilities of the systems during ensemble averaging in the simulations. The systems were then equilibrated for 100 ps using a time-step of 2 fs using first the canonical and later the isothermal-isobaric ensembles. Finally, a ∼1 ns simulation was performed for each λ-value to collect data for the estimation of the free energy contribution. Again the particle Mesh Ewald (PME) algorithm was used for the treatment of non-bonded interactions using a cutoff distance of 12 Å for the Coulombic as well as the van der Waals interactions. A constant temperature was maintained at ∼298 K using the Langevin dynamics integrator with a coupling constant of 1.0 ps. The pressure was controlled using the Parinello-Rahman barostat fixed at 1 bar along with a coupling constant of 1.0 ps and a compressibility value for water set to 4.5 x 10^{-5} bar^{-1} for all simulations. The trajectories obtained

from the AMBER simulation package [1, 10] were converted to GROMACS format for the estimation of the binding free energy through the thermodynamic integration (TI) method. GROMACS 2018.3 [11] was used for all free energy simulations performed after which the energy difference between the two states was estimated using the Benettet Acceptance Ratio (BAR) module of the GROMACS program enabling a straightforward data treatment [12]. The free energy estimates were determined using BAR with bisection, with the precision of the output set and an error estimate taking into account time correlations is made by splitting the data into blocks and determining the free energy differences over those blocks and assuming the blocks are independent. The final error estimate is determined from the average variance over 5 blocks. A range of block numbers for error estimation were also obtained. In general, the results are split in two parts: the last part contains the final results in kJ/mol, together with the error estimate for each part and the total. The first part contains detailed free energy difference estimates and phase space overlap measures in units of kT (together with their computed error estimate).

References

[1] Salomon-Ferrer, R.; Case, D. A.; Walker, R. C. *WIREs Comput. Mol. Sci.* **2013**, *3*(2), 198–210.

[2] Salomon-Ferrer, R.; Gotz, A. W.; Poole, D.; Grand, S. L.; Walker, R. C. *J. Chem. Theory Comput.* **2013**, *9*(9), 3878–3888.

[3] Ryckaert, J.; Ciccotti, G.; Berendsen, H. J. *J. Comput. Phys.* **1977**, *23*(3), 327.

[4] Essmann, U.; Perera, L.; Berkowitz, M. L.; Darden, T.; Lee, H.; Pedersen, L. G. *J. Chem. Phys.* **1995**, *103*(19), 8577–8593.

[5] Roe, D. R.; Cheatham III, T. E. *J. Chem. Theory Comput.* **2013**, *9*(7), 3084–3095.

[6] Humphrey, W.; Dalke, A.; Schulten, K. *J. Mol. Graph.* **1996**, *14*(1), 33.

[7] Pettersen, E. F.; Goddard, T. D.; Huang, C. C.; Couch, G. S.; Greenblatt, D. M.; Meng, E. C.; Ferrin, T. E. UCSF Chimera **2011**, *19*(18), 5507.

[8] Villa, A.; Mark, A. E. *J Comput. Chem.* **2002**, *23*(5), 548–553.

[9] Shirts, M. R.; Pitera, J. W.; Swope, W. C.; Pande, V. S. *J. Chem. Phys.* **2003**, *119*(11), 5740–5761.

[10] Case, D. A.; III, Cheatham III, T. E.; Darden, T.; Gohlke, H.; Luo, R.; Jr, K. M. M.; Onufriev, A.; Simmerling, C.; Wang, B.; Woods, R. J. *J Comput. Chem.* **2005**, *26*(16), 1668–1688.

[11] Abraham, M. J.; Murtola, T.; Schulz, R.; Páll, S.; Smith, J. C.; Hess, B.; Lindahl, E. *SoftwareX* **2015**, *1*, 19–25.

[12] Bennett, C. H. *J. Comput. Phys.* **1976**, *22*(2), 245–268.

Figure S1: Ramachandran plot for the modelled LuxS enzyme obtained from PROCHECK Program

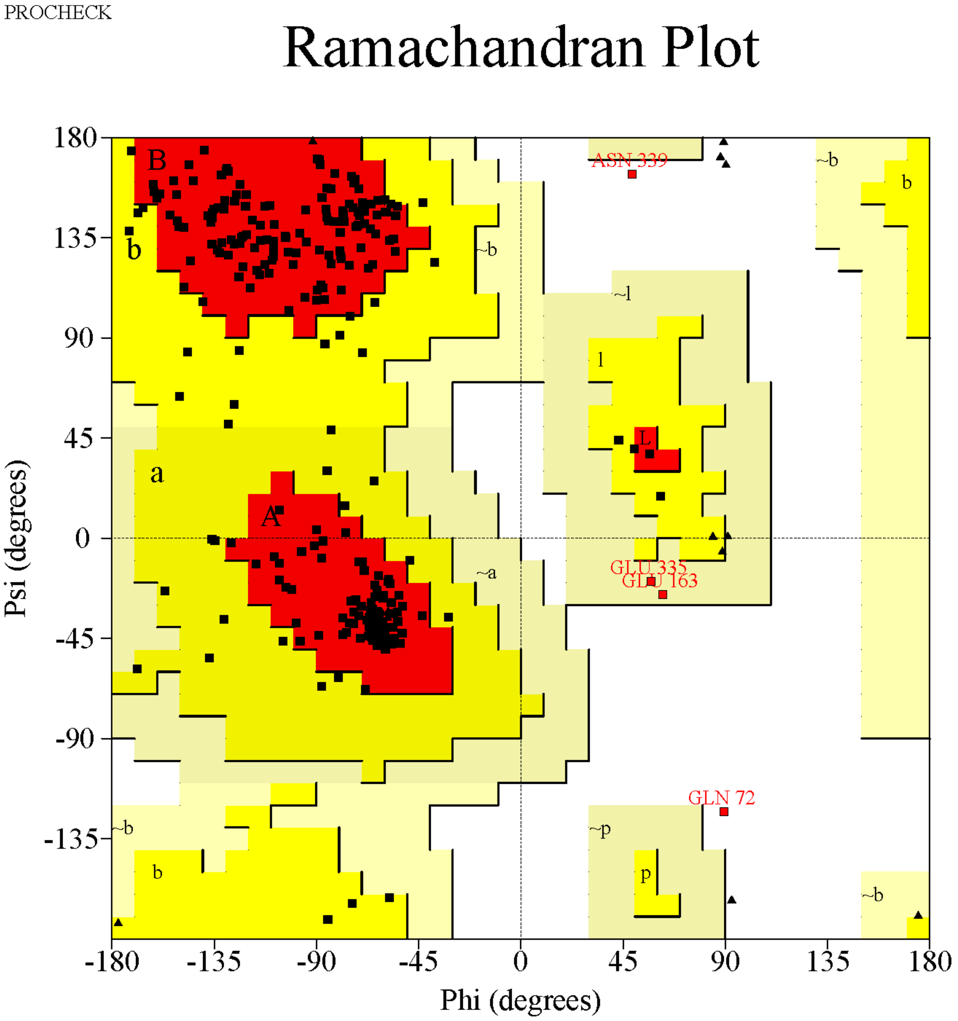

Plot statistics

Residues in most favoured regions [A,B,L]	263	86.2%
Residues in additional allowed regions [a,b,l,p]	38	12.5%
Residues in generously allowed regions [~a,~b,~l,~p]	2	0.7%
Residues in disallowed regions	2	0.7%
	----	------
Number of non-glycine and non-proline residues	305	100.0%
Number of end-residues (excl. Gly and Pro)	7	
Number of glycine residues (shown as triangles)	18	
Number of proline residues	16	

Total number of residues	346	

Based on an analysis of 118 structures of resolution of at least 2.0 Angstroms and R-factor no greater than 20%, a good quality model would be expected to have over 90% in the most favoured regions.

Ligand parameter files obtained from GAFF

RHC inhibitor

---

prepin file

```
    0    0    2

This is a remark line
molecule.res
MOL   XYZ  0
CHANGE     OMIT DU   BEG
  0.0000
   1  DUMM  DU    M       999.000     999.0      -999.0          .00000
   2  DUMM  DU    M       999.000    -999.0       999.0          .00000
   3  DUMM  DU    M      -999.000     999.0       999.0          .00000
   4  O1    o     M         3.261000   -7.261000    4.097000   -0.719564
   5  C1    c     M         2.196000   -7.328000    3.419000    0.668787
   6  O2    o     E         1.932000   -8.041000    2.413000   -0.719564
   7  C2    c3    M         1.079000   -6.384000    3.894000    0.211496
   8  N1    n4    3         1.039000   -5.089000    3.121000   -0.577087
   9  H1    hn    E         0.779000   -5.285000    2.145000    0.364494
  10  H2    hn    E         0.310000   -4.469000    3.503000    0.364494
  11  H17   hn    E         1.926000   -4.632000    3.189000    0.364494
  12  H3    hx    E         1.262000   -6.093000    4.936000    0.022014
  13  C3    c3    M        -0.326000   -6.993000    3.778000   -0.163932
  14  H4    hc    E        -0.542000   -7.269000    2.737000    0.064226
  15  H5    hc    E        -1.057000   -6.219000    4.044000    0.064226
  16  C4    c3    M        -0.502000   -8.223000    4.671000    0.094729
  17  H6    h1    E         0.001000   -9.088000    4.228000    0.082610
  18  H7    h1    E        -0.072000   -8.067000    5.667000    0.082610
```

19	S1	ss	M	-2.268000	-8.637000	4.850000	-0.241420
20	C5	c3	M	-2.718000	-7.395000	6.113000	-0.526807
21	H8	h1	E	-2.503000	-7.832000	7.094000	0.160840
22	H9	h1	E	-2.080000	-6.519000	5.986000	0.160840
23	C6	c3	M	-4.200000	-7.002000	6.006000	0.591398
24	O3	oh	S	-4.985000	-8.078000	6.502000	-0.620803
25	H10	ho	E	-4.802000	-8.169000	7.453000	0.415802
26	H11	h1	E	-4.460000	-6.847000	4.952000	0.102413
27	C7	c3	M	-4.480000	-5.702000	6.787000	-0.004201
28	O4	oh	S	-3.671000	-4.677000	6.188000	-0.660131
29	H12	ho	E	-3.710000	-3.896000	6.765000	0.425536
30	H13	h1	E	-4.136000	-5.813000	7.824000	0.042683
31	C8	c3	M	-5.930000	-5.177000	6.792000	0.405812
32	O5	oh	S	-6.538000	-5.376000	5.521000	-0.734700
33	H14	ho	E	-7.421000	-4.970000	5.552000	0.466893
34	H15	h1	E	-5.895000	-4.091000	6.948000	-0.021687
35	C9	c	M	-6.816000	-5.750000	7.893000	0.162232
36	H16	h4	E	-6.320000	-5.675000	8.864000	0.068791
37	O6	o	M	-7.158000	-7.107000	7.655000	-0.397522

LOOP

IMPROPER

C2 O1 C1 O2

C8 H16 C9 O6

DONE

STOP

frcmod file

Remark line goes here

MASS

o 16.000   0.434

c 12.010   0.616

c3 12.010   0.878

n4 14.010   0.530

hn 1.008   0.161

hx 1.008   0.135

hc 1.008   0.135

h1 1.008   0.135

ss 32.060   2.900

oh 16.000   0.465

ho 1.008   0.135

h4 1.008   0.135

BOND

c -o   637.70   1.218

c -c3 313.00   1.524

c3-n4 283.30   1.511

c3-hx 338.70   1.091

c3-c3 300.90   1.538

hn-n4 373.20   1.030

c3-hc 330.60   1.097

c3-h1 330.60   1.097

c3-ss 215.90   1.839

c3-oh 316.70   1.423

ho-oh 371.40   0.973

c -h4 310.70   1.112

ANGLE

o -c -o 77.900 130.250

c3-c -o 67.400 123.200

c -c3-n4 65.500 110.730

c -c3-hx 46.900 108.850

c -c3-c3 63.300 111.040

c3-n4-hn 45.900 110.110

c3-c3-hc 46.300 109.800

c3-c3-c3 62.900 111.510

hx-c3-n4 48.600 108.010

c3-c3-n4 64.200 114.210

hn-n4-hn 40.600 108.300

c3-c3-hx 46.200 110.560

c3-c3-h1 46.400 109.560

c3-c3-ss 61.300 110.270

hc-c3-hc 39.400 107.580

c3-ss-c3 60.200 99.240

h1-c3-h1 39.200 108.460

h1-c3-ss 42.100 108.760

c3-c3-oh 67.500 110.190

c3-oh-ho 47.400 107.260

h1-c3-oh 50.900 110.260

c3-c -h4 45.600 114.640

c -c3-oh 68.300 108.790

c -c3-h1 47.000 108.220

h4-c -o 54.200 120.700

DIHE

o -c -c3-n4 6 0.000 180.000 2.000

o -c -c3-hx 6 0.000 180.000 2.000

o -c -c3-c3	6	0.000	180.000	2.000
c -c3-n4-hn	9	1.400	0.000	3.000
c -c3-c3-hc	9	1.400	0.000	3.000
c -c3-c3-c3	9	1.400	0.000	3.000
c3-c3-c3-h1	9	1.400	0.000	3.000
c3-c3-c3-ss	9	1.400	0.000	3.000
hc-c3-c3-n4	9	1.400	0.000	3.000
c3-c3-c3-n4	9	1.400	0.000	3.000
hx-c3-n4-hn	9	1.400	0.000	3.000
c3-c3-n4-hn	9	1.400	0.000	3.000
hc-c3-c3-hx	9	1.400	0.000	3.000
c3-c3-c3-hx	9	1.400	0.000	3.000
c3-c3-ss-c3	3	1.000	0.000	3.000
h1-c3-c3-hc	9	1.400	0.000	3.000
hc-c3-c3-ss	9	1.400	0.000	3.000
h1-c3-ss-c3	3	1.000	0.000	3.000
oh-c3-c3-ss	9	1.400	0.000	3.000
h1-c3-c3-ss	9	1.400	0.000	3.000
c3-c3-oh-ho	1	0.160	0.000	-3.000
c3-c3-oh-ho	1	0.250	0.000	1.000
c3-c3-c3-oh	9	1.400	0.000	3.000
c3-c3-c3-c3	1	0.180	0.000	-3.000
c3-c3-c3-c3	1	0.250	180.000	-2.000
c3-c3-c3-c3	1	0.200	180.000	1.000
h1-c3-c3-oh	1	0.000	0.000	-3.000
h1-c3-c3-oh	1	0.250	0.000	1.000
h1-c3-c3-h1	9	1.400	0.000	3.000
oh-c3-c3-oh	1	0.144	0.000	-3.000
oh-c3-c3-oh	1	1.175	0.000	2.000
h1-c3-oh-ho	3	0.500	0.000	3.000

h4-c -c3-c3   6   0.000   180.000   2.000

c -c3-c3-oh   9   1.400   0.000   3.000

c -c3-c3-h1   9   1.400   0.000   3.000

h4-c -c3-oh   6   0.000   180.000   2.000

o -c -c3-oh   6   0.000   180.000   2.000

c -c3-oh-ho   3   0.500   0.000   3.000

h4-c -c3-h1   6   0.000   180.000   2.000

o -c -c3-h1   1   0.800   0.000   -1.000

o -c -c3-h1   1   0.000   0.000   -2.000

o -c -c3-h1   1   0.080   180.000   3.000

IMPROPER

c3-o -c -o   1.1   180.0   2.0   Using general improper torsional angle X- o- c- o, penalty score= 3.0)

c3-h4-c -o   10.5   180.0   2.0   Using general improper torsional angle X- X- c- o, penalty score= 6.0)

NONBON

o   1.6612 0.2100

c   1.9080 0.0860

c3   1.9080 0.1094

n4   1.8240 0.1700

hn   0.6000 0.0157

hx   1.1000 0.0157

hc   1.4870 0.0157

h1   1.3870 0.0157

ss   2.0000 0.2500

oh   1.7210 0.2104

ho   0.0000 0.0000

h4   1.4090 0.0150

KRI inhibitor

---

prepin file

```
    0    0    2

This is a remark line
molecule.res
MOL   XYZ  0
CHANGE     OMIT DU   BEG
  0.0000
   1  DUMM  DU    M       999.000     999.0      -999.0          .00000
   2  DUMM  DU    M       999.000    -999.0       999.0          .00000
   3  DUMM  DU    M      -999.000     999.0       999.0          .00000
   4  O1    o     M        47.433000   36.726000   47.275000   -0.741706
   5  C1    c     M        46.526000   37.474000   47.573000    0.761110
   6  O2    o     E        46.467000   38.658000   47.287000   -0.741706
   7  C2    c3    M        45.862000   37.164000   48.934000    0.325018
   8  N1    n4    3        46.303000   38.209000   49.982000   -0.790951
   9  H1    hn    E        47.321000   38.118000   50.127000    0.415689
  10  H2    hn    E        46.182000   39.132000   49.516000    0.415689
  11  H3    hn    E        45.798000   38.159000   50.871000    0.415689
  12  H4    hx    E        46.261000   36.191000   49.229000    0.007682
  13  C3    c3    M        44.326000   37.048000   48.783000   -0.311195
  14  H5    hc    E        44.103000   36.366000   47.950000    0.039805
  15  H6    hc    E        43.897000   38.020000   48.505000    0.039805
  16  C4    c3    M        43.650000   36.498000   50.057000    0.594536
  17  H7    h1    E        44.110000   35.536000   50.319000   -0.125855
  18  H8    h1    E        43.773000   37.185000   50.902000   -0.125855
  19  S1    ss    M        41.858000   36.196000   49.763000   -0.257505
  20  C5    c3    M        41.117000   37.869000   50.028000   -0.195765
```

21	H9	h1	E	41.723000	38.624000	49.516000	0.139804
22	H10	h1	E	40.118000	37.876000	49.571000	0.139804
23	C6	c3	M	40.976000	38.217000	51.531000	0.403623
24	O3	oh	S	40.031000	37.334000	52.141000	-0.707418
25	H11	ho	E	39.290000	37.869000	52.499000	0.440148
26	H12	h1	E	41.952000	38.059000	52.006000	-0.064771
27	C7	c3	M	40.571000	39.699000	51.755000	-0.001443
28	O4	oh	S	39.456000	40.069000	50.917000	-0.657221
29	H13	ho	E	38.640000	40.021000	51.467000	0.445226
30	H14	h1	E	41.429000	40.332000	51.498000	0.094620
31	C8	c	M	40.229000	39.960000	53.232000	0.681652
32	O5	o	E	39.125000	39.679000	53.667000	-0.578321
33	N2	n	M	41.262000	40.388000	54.049000	-0.434031
34	H15	hn	E	42.187000	40.580000	53.687000	0.347394
35	O6	oh	M	41.031000	40.759000	55.402000	-0.397052
36	H16	ho	E	40.768000	39.922000	55.843000	0.423501

LOOP

IMPROPER

C2 O1 C1 O2

C7 N2 C8 O5

C8 H15 N2 O6

DONE

STOP

frcmod file

Remark line goes here

MASS

```
o  16.000        0.434
c  12.010        0.616
c3 12.010        0.878
n4 14.010        0.530
hn 1.008         0.161
hx 1.008         0.135
hc 1.008         0.135
h1 1.008         0.135
ss 32.060        2.900
oh 16.000        0.465
ho 1.008         0.135
n  14.010        0.530
```

BOND

```
c -o   637.70   1.218
c -c3  313.00   1.524
c3-n4  283.30   1.511
c3-hx  338.70   1.091
c3-c3  300.90   1.538
hn-n4  373.20   1.030
c3-hc  330.60   1.097
c3-h1  330.60   1.097
c3-ss  215.90   1.839
c3-oh  316.70   1.423
ho-oh  371.40   0.973
c -n   427.60   1.379
hn-n   403.20   1.013
```

n -oh 400.20   1.406

ANGLE

o -c -o   77.900   130.250

c3-c -o   67.400   123.200

c -c3-n4   65.500   110.730

c -c3-hx   46.900   108.850

c -c3-c3   63.300   111.040

c3-n4-hn   45.900   110.110

c3-c3-hc   46.300   109.800

c3-c3-c3   62.900   111.510

hx-c3-n4   48.600   108.010

c3-c3-n4   64.200   114.210

hn-n4-hn   40.600   108.300

c3-c3-hx   46.200   110.560

c3-c3-h1   46.400   109.560

c3-c3-ss   61.300   110.270

hc-c3-hc   39.400   107.580

c3-ss-c3   60.200   99.240

h1-c3-h1   39.200   108.460

h1-c3-ss   42.100   108.760

c3-c3-oh   67.500   110.190

c3-oh-ho   47.400   107.260

h1-c3-oh   50.900   110.260

c3-c -n   66.800   115.180

c -c3-oh   68.300   108.790

c -c3-h1   47.000   108.220

c -n -hn   48.300   117.550

c -n -oh   68.200   115.510

n -c -o   74.200   123.050

ho-oh-n   50.500   101.290

hn-n -oh   50.400   110.740

DIHE

o -c -c3-n4   6   0.000   180.000   2.000

o -c -c3-hx   6   0.000   180.000   2.000

o -c -c3-c3   6   0.000   180.000   2.000

c -c3-n4-hn   9   1.400   0.000   3.000

c -c3-c3-hc   9   1.400   0.000   3.000

c -c3-c3-c3   9   1.400   0.000   3.000

c3-c3-c3-h1   9   1.400   0.000   3.000

c3-c3-c3-ss   9   1.400   0.000   3.000

hc-c3-c3-n4   9   1.400   0.000   3.000

c3-c3-c3-n4   9   1.400   0.000   3.000

hx-c3-n4-hn   9   1.400   0.000   3.000

c3-c3-n4-hn   9   1.400   0.000   3.000

hc-c3-c3-hx   9   1.400   0.000   3.000

c3-c3-c3-hx   9   1.400   0.000   3.000

c3-c3-ss-c3   3   1.000   0.000   3.000

h1-c3-c3-hc   9   1.400   0.000   3.000

hc-c3-c3-ss   9   1.400   0.000   3.000

h1-c3-ss-c3   3   1.000   0.000   3.000

oh-c3-c3-ss   9   1.400   0.000   3.000

h1-c3-c3-ss   9   1.400   0.000   3.000

c3-c3-oh-ho   1   0.160   0.000   -3.000

c3-c3-oh-ho   1   0.250   0.000   1.000

c3-c3-c3-oh   9   1.400   0.000   3.000

h1-c3-c3-oh   1   0.000   0.000   -3.000

h1-c3-c3-oh   1   0.250   0.000   1.000

h1-c3-c3-h1   9   1.400   0.000   3.000

n -c -c3-c3   1   0.100   0.000   -4.000

n -c -c3-c3   1   0.070   0.000   2.000

oh-c3-c3-oh   1   0.144   0.000   -3.000

oh-c3-c3-oh   1   1.175   0.000   2.000

c -c3-c3-oh   9   1.400   0.000   3.000

h1-c3-oh-ho   3   0.500   0.000   3.000

c -c3-c3-h1   9   1.400   0.000   3.000

c3-c -n -hn   4   10.000   180.000   2.000

c3-c -n -oh   4   10.000   180.000   2.000

o -c -c3-oh   6   0.000   180.000   2.000

n -c -c3-oh   6   0.000   180.000   2.000

c -c3-oh-ho   3   0.500   0.000   3.000

o -c -c3-h1   1   0.800   0.000   -1.000

o -c -c3-h1   1   0.000   0.000   -2.000

o -c -c3-h1   1   0.080   180.000   3.000

n -c -c3-h1   6   0.000   180.000   2.000

c -n -oh-ho   2   3.000   0.000   2.000

o -c -n -hn   1   2.500   180.000   -2.000

o -c -n -hn   1   2.000   0.000   1.000

o -c -n -oh   4   10.000   180.000   2.000

hn-n -oh-ho   2   3.000   0.000   2.000

IMPROPER

c3-o -c -o   1.1   180.0   2.0   Using general improper torsional angle X- o- c- o, penalty score= 3.0)

c3-n -c -o   10.5   180.0   2.0   Using general improper torsional angle X- X- c- o, penalty score= 6.0)

c -hn-n -oh   1.1   180.0   2.0   Using the default value

NONBON

o   1.6612 0.2100

c	1.9080	0.0860
c3	1.9080	0.1094
n4	1.8240	0.1700
hn	0.6000	0.0157
hx	1.1000	0.0157
hc	1.4870	0.0157
h1	1.3870	0.0157
ss	2.0000	0.2500
oh	1.7210	0.2104
ho	0.0000	0.0000
n	1.8240	0.1700
